# Neuropeptides Modulate Female Chemosensory Processing upon Mating in *Drosophila*

**DOI:** 10.1371/journal.pbio.1002455

**Published:** 2016-05-04

**Authors:** Ashiq Hussain, Habibe K. Üçpunar, Mo Zhang, Laura F. Loschek, Ilona C. Grunwald Kadow

**Affiliations:** Max-Planck Institute of Neurobiology, Sensory Neurogenetics Research Group, Martinsried, Germany; Vlaams Instituut voor Biotechnologie and Katholieke Universiteit Leuven, BELGIUM

## Abstract

A female’s reproductive state influences her perception of odors and tastes along with her changed behavioral state and physiological needs. The mechanism that modulates chemosensory processing, however, remains largely elusive. Using *Drosophila*, we have identified a behavioral, neuronal, and genetic mechanism that adapts the senses of smell and taste, the major modalities for food quality perception, to the physiological needs of a gravid female. Pungent smelling polyamines, such as putrescine and spermidine, are essential for cell proliferation, reproduction, and embryonic development in all animals. A polyamine-rich diet increases reproductive success in many species, including flies. Using a combination of behavioral analysis and in vivo physiology, we show that polyamine attraction is modulated in gravid females through a G-protein coupled receptor, the sex peptide receptor (SPR), and its neuropeptide ligands, MIPs (myoinhibitory peptides), which act directly in the polyamine-detecting olfactory and taste neurons. This modulation is triggered by an increase of SPR expression in chemosensory neurons, which is sufficient to convert virgin to mated female olfactory choice behavior. Together, our data show that neuropeptide-mediated modulation of peripheral chemosensory neurons increases a gravid female’s preference for important nutrients, thereby ensuring optimal conditions for her growing progeny.

## Abstract 2

During pregnancy, women frequently report that their food preferences change—sometimes dramatically. This study unravels a mechanism that changes a mated female’s perception of odors and tastes and thereby adapts her food choices to her reproductive state.

## Author Summary

Food choices often correlate with nutritional needs or physiological states of an animal. For instance, during pregnancy, women frequently report that their food preferences change—sometimes dramatically. In part, this change in preference is brought about by a change in the perception of smells and tastes. Research has shown that female insects also change their food and egg-laying site preferences depending on their reproductive state. However, the mechanisms that trigger these changes are not understood in either mammals or insects. We have unraveled a mechanism that changes a mated female’s perception of odors and tastes and thereby adapts her choices to her reproductive state. Using the model fly *Drosophila melanogaster*, we show that mating increases females’ interest in sources of specific beneficial nutrients: polyamines such as spermine and putrescine. Polyamine levels in the body are maintained by diet, microorganisms in the gut, and own synthesis. Increased levels are required during pregnancy and reproduction. Indeed, mated females were more attracted to the taste and smell of polyamines than virgins were. We found that this behavioral modulation is regulated through a secreted peptide and its receptor, whose expression rises markedly in sensory organs upon mating. This signal appears to change the intensity of how polyamine taste or smell information reaches the brain and ultimately elicits a choice. Given that odor and taste processing in mammals and insects are similar, our findings in flies can lead to a better understanding of how dynamic physiological states affect our perception of the environment and lead us to adapt our choices of food and other relevant decisions.

## Introduction

The behavior of females in most animal species changes significantly as a consequence of mating. Those changes are interpreted from an evolutionary standpoint as the female’s preparation to maximize the fitness of her offspring. In general, they entail a qualitative and quantitative change in her diet, as well as the search for an optimal site where her progeny will develop. In humans, the eating behavior and perception of tastes and odors of a pregnant woman are modulated in concert with altered physiology and the specific needs of the embryo [1–3]. While several neuromodulatory molecules such as noradrenaline are found in the vertebrate olfactory and gustatory systems, little is known about how reproductive state and pregnancy shape a female’s odor and taste preferences [4,5]. Very recent work in the mouse showed that olfactory sensory neurons (OSNs) are modulated during the estrus cycle [6]. Progesterone receptor expressed in OSNs decreases the sensitivity of pheromone-detecting OSNs and thereby reduces the non-sexually receptive female’s interest in male pheromones. The mechanisms of how mating, pregnancy, and lactation shape the response of the female olfactory and gustatory systems remain poorly understood.

The neuronal underpinnings of mating and its consequences on female behaviors have arguably been best characterized in the fruit fly *Drosophila melanogaster* [7,8]. Shortly after copulation, female flies engage in a series of post-mating behaviors contrasting with those of virgins: their sexual receptivity decreases, and they feed to accumulate essential resources needed for the production of eggs [9–12]; finally, they lay their eggs. This suite of behaviors results from a post-mating trigger located in the female’s reproductive tract [12]. Sensory neurons extending their dendrites directly into the oviduct are activated by a component of the male’s ejaculate, the sex peptide (SP) [13,14]. Sex peptide receptor (SPR) expressed by these sensory neurons triggers the post-mating switch [15]. Mated females mutant for *SPR* produce and lay fewer eggs while maintaining a high sexual receptivity [13–15]. In addition to SP, male ejaculate contains more than 200 proteins, which are transferred along with SP into the female. These have been implicated in conformational changes of the uterus, induction of ovulation, and sperm storage [7,16–18].

Additional SPR ligands have been identified that are not required for the canonical post-mating switch, opening the possibility that this receptor is involved in the neuromodulation of other processes [19–22]. These alternative ligands, the myoinhibitory peptides (MIPs)/allatostatin-Bs, unlike SP, have been found outside of drosophilids, in many other insect species such as the silkmoth (*Bombyx mori*), several mosquito species, and the red flour beetle (*Tribolium castaneum*) [19]. They are expressed in the brain of flies and mosquitoes, including in the centers of olfactory and gustatory sensory neuron projections, the antennal lobe (AL), and the subesophageal zone (SEZ), respectively [19,23,24]. Although these high-affinity SPR ligands have recently been implicated in the control of sleep in *Drosophila* males and females [25], nothing thus far suggests a function in reproductive behaviors [19].

To identify optimal food and oviposition sites, female flies rely strongly on their sense of smell and taste [26–29]. *D*. *melanogaster* females prefer to oviposit in decaying fruit and use byproducts of fermentation such as ethanol and acetic acid to choose oviposition sites [29,30]. Their receptivity to these byproducts is enhanced by their internal state [29,31]. It was shown, for instance, that the presence of an egg about to be laid results in increased attraction to acetic acid [31]. Yet the mechanisms linking reproductive state to the modulation of chemosensory processing remain unknown.

We have examined the causative mechanisms that integrate reproductive state into preference behavior and chemosensory processing. We have focused on the perception of another class of byproducts of fermenting fruits, polyamines. Polyamines such as putrescine, spermine, and spermidine are important nutrients that are associated with reproductive success across animal species [32]. A diet high in polyamines indeed increases the number of offspring of a fly couple, and female flies prefer to lay their eggs on polyamine-rich food [33]. Importantly, we have previously characterized the chemosensory mechanisms flies use to find and evaluate polyamine-rich food sources and oviposition sites. In brief, volatile polyamines are detected by OSNs on the fly’s antenna, co-expressing two ionotropic receptors (IRs), IR41a and IR76b [33,34]. Interestingly, the taste of polyamines is also detected by IR76b in labellar gustatory receptor neurons (GRNs) [33].

This beneficial role of polyamines has a well-characterized biological basis: polyamines are essential for basic cellular processes such as cell growth and proliferation, and are of specific importance during reproduction [35]. They enhance the quality of sperm and egg and are critical during embryogenesis and postnatal development [32,36]. While the organism can generate polyamines, a significant part is taken in with the diet [37,38]. Moreover, endogenous synthesis of polyamines declines with ageing and can be compensated for through a polyamine-rich diet [32]. Therefore, these compounds represent a sensory cue as well as an essential component of the diet of a gravid female fly.

Here, we show that the olfactory and gustatory perception of polyamines is modulated by the female’s reproductive state and guides her choice behavior accordingly. This sensory and behavioral modulation depends on SPR and its conserved ligands, the MIPs that act directly on the chemosensory neurons themselves. Together, our results suggest that mating-state-dependent neuropeptidergic modulation of chemosensory neurons matches the female fly’s decision-making to her physiological needs.

## Results

### Mating State Modulates the Perception of Polyamines

**Fig 1:**
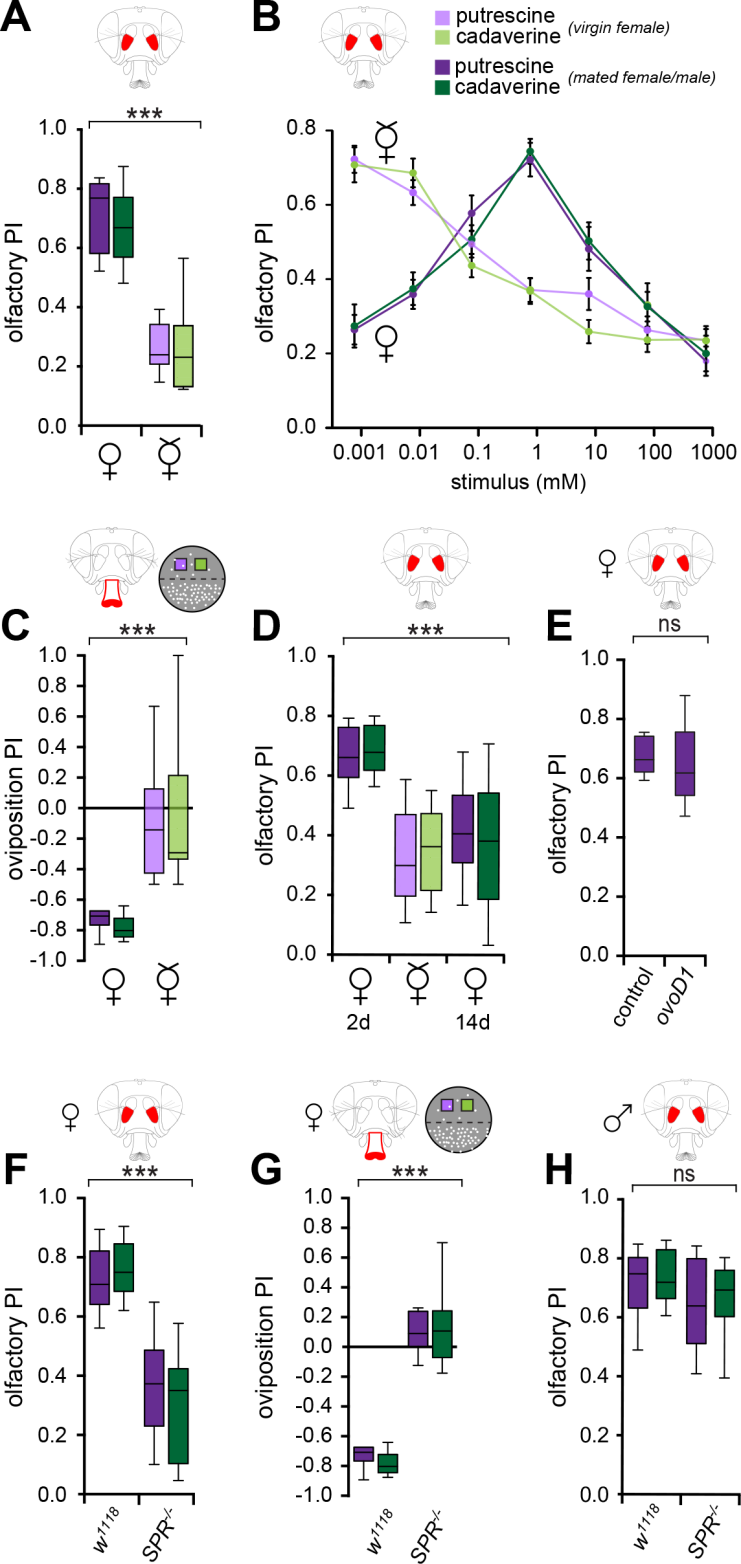
Mating state modulates the perception of polyamines. (A) Virgin flies are less attracted to a high polyamine concentration of 1 mM (10 ppm) as compared to mated flies. Olfactory preference index of Canton S mated (♀) and Canton S virgin (☿) females in the T-maze assay. Violet and green bars represent putrescine and cadaverine, respectively. (*n* = 8, 60 mated (♀) or virgin (☿) flies/trial). (B) Mated females, unlike virgins, preferred relatively high concentrations of polyamines, naturally present in fermenting fruit (1 mM or 10ppm). By contrast, virgin females were most attracted to very low concentrations of polyamine. Line graph shows dose-dependent olfactory preference index of mated (♀) and virgin (☿) flies to polyamines. (*n* = 8 ± SEM, 60 mated (♀) or virgin (☿) flies/trial). (C) Virgin flies show no preference between polyamines (putrescine or cadaverine, 1 mM) and 1% low melting agarose, and deposited their low number of eggs on either site of the assay. (*n* = 8, 60 mated (♀) or virgin (☿) flies/trial). (D) Polyamine preference appears to correlate with the female’s egg-laying activity. Graphs show olfactory preference index of females 2 d post-mating (♀, 2 d), virgin females (☿), and females 14 d post-mating (♀, 14 d) to 10 ppm of polyamine. (*n* = 8, 60 mated (♀, 2 d), virgin (☿) and mated (♀, 14 d) female flies/trial). (E) Mated but sterile *ovoD1* mutant females (*ovoD1/+*, Canton S) show similar attraction to polyamine odor compared to wildtype controls (*+/+*, Canton S). (F) Mated sex peptide receptor mutant (*SPR*^*-/-*^*)* female flies display a significantly reduced attraction to polyamine odor (*n* = 8, 60 flies/trial). (G) Oviposition preference index of mated sex peptide receptor mutant (*SPR*^*-/-*^) females. Mated *SPR* mutant females show indifference to polyamines. (n = 8, 60 mated (♀) flies/ trial). (H) Olfactory preference for 10 ppm polyamine of *SPR*^*-/-*^ male flies is comparable to control males. Box plots show median and upper/lower quartiles (*n* = 8, 60 flies/trial). All *p*-values were calculated via two-way ANOVA with the Bonferroni multiple comparison post-hoc test, with the exception of (E), where p-values were calculated with an unpaired t-test (ns > 0.05, *p ≤ 0.05, **p ≤ 0.01, ***p ≤ 0.001).

Males and female flies are strongly attracted to polyamines [33]. The perception of sensory stimuli, however, can be modulated and depends on behavioral context [39]. Given that polyamine-rich foods increase the number of progeny [33], we wondered whether mating state influences the perception of these important molecules. To test this, we compared olfactory and oviposition behaviors of mated to virgin female flies. In an olfactory choice assay, the T-maze, mated females showed a strong attraction to volatile polyamines, which requires their sense of smell, as we have shown in the companion paper and as previously suggested by Silbering et al. [33,34]. Virgin flies displayed a significantly altered preference for the polyamines putrescine and cadaverine compared to mated flies (Fig 1A). While mated females preferred relatively high concentrations of polyamines typically present in fermenting fruit (1 mM or 10 ppm, [36,37]), virgin females showed strong attraction to only the lowest levels and increasing avoidance of higher levels of these odors (Fig 1B).

We next analyzed whether virgin flies would make different egg-laying choices compared to mated flies. Mated females taste polyamines with taste sensilla on their labellum and use this information during egg-laying decisions [33]. Although egg-laying substrates containing just polyamines are avoided as egg-laying substrates because of their bitter taste, polyamine-rich sugary substrates such as decaying fruit are strongly preferred over fresh fruit [33]. To assay the egg-laying preferences, we used a simple oviposition assay consisting of a plate with a plain agarose substrate (1%) that was on one-half of the egg-laying plate supplemented with the polyamines, putrescine or cadaverine (1 mM, Fig 1C, see Materials and Methods). Consistent with our dissection of polyamine perception [33], mated flies displayed a strong preference and laid the majority of their eggs on plain agarose (Fig 1C). By contrast, virgin females, albeit laying very few (and unfertilized) eggs, distributed their eggs equally between polyamine and control sides (Figs 1C and S1A). Therefore, we concluded that, while mated females actively develop a choice behavior, virgin females are indifferent to polyamines as an egg-laying substrate. Taken together, odor as well as taste perception of polyamines strongly depends on the female fly’s mating state.

We have shown that a polyamine-rich diet increases the number of offspring of a fly couple [33]. These data could potentially indicate that needs arising through egg production and laying, and not exclusively or primarily through mating, drive a female to seek polyamines. We therefore first tested whether polyamine choice behavior correlated with the female’s egg-laying activity and time after mating. This appeared to be the case, because mated females that had ceased to lay eggs at 14 d after mating returned to their pre-mating preference behavior and made choices that resembled the choices of virgin flies (Fig 1D). This return to virgin behavior could be due to the time elapsed after mating or to a reduction in egg-laying. To dissect the relative contribution of egg-laying activity and mating, we analyzed the preference behavior of mated *ovoD1* mutant females [40]. These females are sterile due to an atrophy of the ovaries. Mated *ovoD1* mutant females showed the same preference to polyamines in the T-maze compared to control mated females (Fig 1E). From these data, it appears that mating itself provides a key signal that changes the female’s perception and stimulates her to seek polyamines.

While previous research has shown that mating state and egg-laying activity influence the choice behavior of female flies when selecting food or oviposition substrates [9,29,31], how mating state modulates neural sensitivity and processing of sensory information remains not understood. Having defined the gustatory and olfactory receptors and sensory neurons for the detection of polyamines [33], we sought to identify the mechanism that modulates this detection and processing in a mating state-dependent manner. SPR and SP are required for the classical post-mating switch (see Introduction) and changes in feeding behavior [9,10,41]. To test the role of SPR in mating-state-dependent polyamine choice behavior, we initially examined the olfactory preference and oviposition behavior of *SPR* mutant females (*Df(1)Exel6234*) [15]. Mated *SPR* mutant females showed a significantly reduced preference behavior in the T-maze (odor) as well as in oviposition assays (taste) compared to that of mated control females (Fig 1F and 1G). Importantly, *SPR* mutant males maintained the same level of attraction as wildtype control males, possibly representing the constant need of polyamines such as spermine and spermidine for sperm production (Fig 1H). These results indicated that the SPR pathway is part of the mechanism that controls mating-induced changes in the perception of the smell and taste of polyamines.

### G-Protein Coupled Receptor (GPCR) Signaling in Chemosensory Neurons Modulates Female Perception

**Fig 2:**
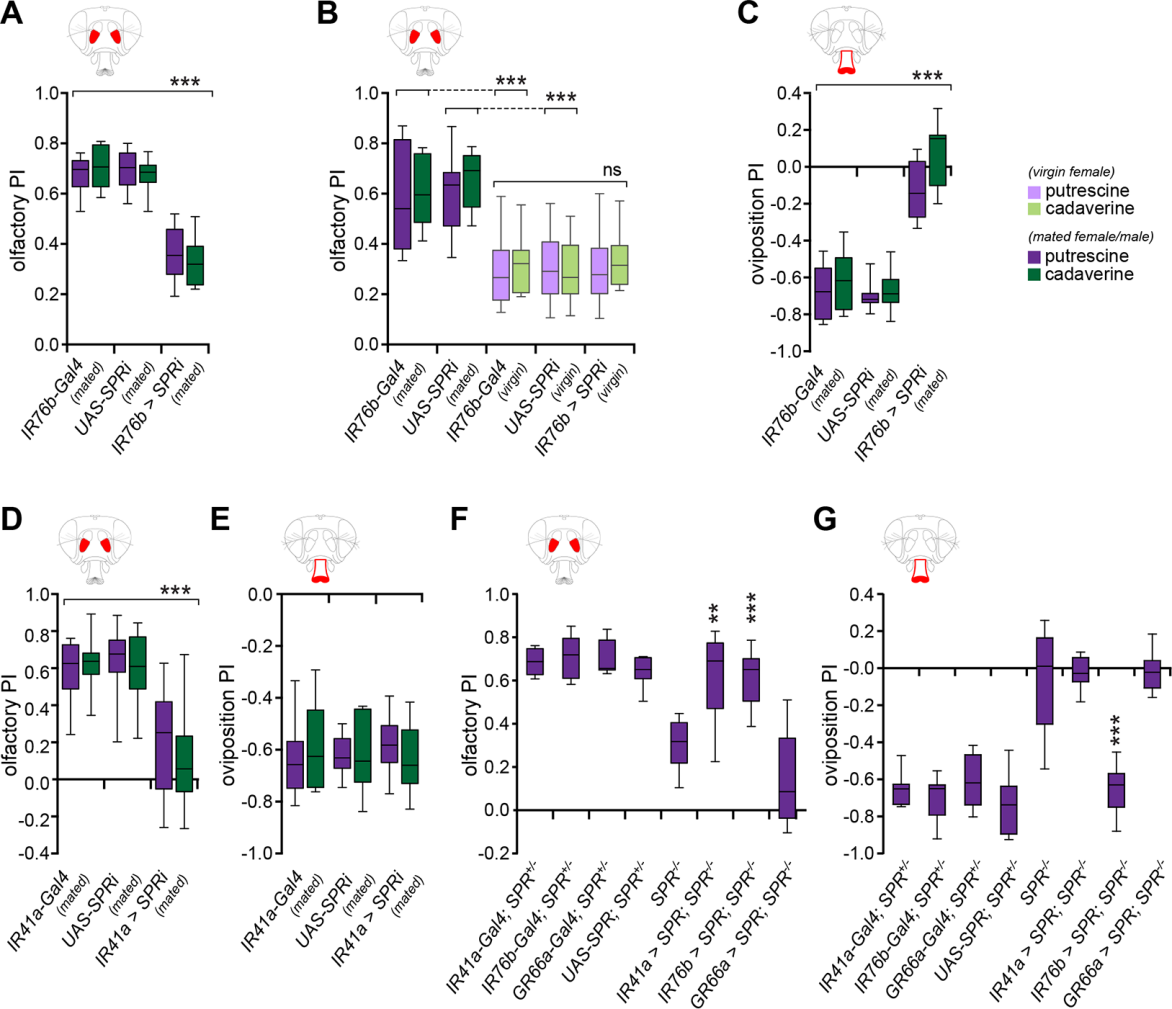
GPCR signaling in chemosensory neurons modulates female perception. (A) Knockdown of *SPR* in IR76b polyamine chemosensory neurons using RNAi (*IR76b-Gal4;UAS-SPRi*) significantly reduces olfactory preference to 10 ppm putrescine or cadaverine in mated females as compared to mated controls. (*n* = 8, 60 mated (♀) flies/trial). (B) The effect of SPRi in IR76b neurons is mating state-dependent, as knockdown of *SPR* (*IR76b-Gal4;UAS-SPRi*) does not further decrease the olfactory attraction of virgin females to polyamines compared to control virgins. (*n* = 8, 60 mated (♀) or virgin (☿) flies/trial). (C) Oviposition avoidance of a 1 mM polyamine/agarose substrate compared to a plain agarose substrate is strongly reduced upon knockdown of *SPR* in IR76b neurons (*IR76b-Gal4;UAS-SPRi*) (*n* = 8, 60 mated (♀) flies/trial). (D) Knockdown of *SPR* in IR41a neurons (*IR41a-Gal4;UAS-SPRi*) leads to a similar decrease in attraction to the odor of polyamine (10 ppm) in the T-maze as compared to knockdown of *SPR* with IR76b-Gal4, suggesting that SPR is required in olfactory neurons to enhance the attraction of mated females to the polyamine odors (*n* = 8, 60 mated (♀) flies/trial). (E) Knockdown of *SPR* in IR41a neurons (*IR41a-Gal4;UAS-SPRi*) did not affect oviposition behavior, and female behavior remained like their genetic controls. This result is consistent with the lack of expression of IR41a in taste neurons. (F) Re-expression of SPR using either IR41a-Gal4 or IR76b-Gal4 neurons fully rescued the *SPR* mutant phenotype of mated females in olfaction behavior to 10 ppm polyamines. (*n* = 8, 60 flies/trial). (G) Re-expression of SPR in IR76b taste neurons using IR76b-Gal4 fully rescued the *SPR* mutant phenotype of mated females in oviposition behavior. Conversely, re-expression of SPR in IR41a olfactory neurons or GR66a bitter taste neurons did not rescue oviposition preference behavior. (*n* = 8, 60 flies/trial). Box plots show median and upper/lower quartiles. All *p*-values were calculated via two-way ANOVA with the Bonferroni multiple comparison post-hoc test (ns > 0.05, *p ≤ 0.05, **p ≤ 0.01, ***p ≤ 0.001).

Increasing evidence in different model organisms indicates that chemosensory neurons themselves are potent targets for neuromodulation [6,42–44]. Although SPR is required in specific internal sensory neurons in the female reproductive tract for the canonical post-mating switch, its rather broad expression in the nervous system, including chemosensory organs and their projection zones in the brain [15,45], prompted us to ask whether SPR signaling was acting directly in peripheral chemosensory neurons. Previous work successfully employed RNA interference (RNAi) directed against *SPR* to identify the set of sensory neurons in the female reproductive tract sufficient to trigger two important post-mating behaviors: increased egg-laying and rejection of males [13,14]. We induced RNAi against *SPR* (*UAS-SPRi*) specifically in olfactory and gustatory neurons that sense polyamines, using the driver IR76b-Gal4 [33]. Importantly, this driver was not expressed in the internal sensory neurons that require SPR to induce the mating switch (S2A and S2C Fig). Mated females of the genotype *IR76b-Gal4;UAS-SPRi* showed a significantly reduced attraction to polyamine odor in the T-maze assay as compared to controls (Fig 2A). Remarkably, this reduction was similar to the reduction seen in *SPR* mutants (see Fig 1). Importantly, *SPR* RNAi did not reduce the attraction of virgin females further, showing that the regulation by SPR is indeed mating-state-dependent (Fig 2B). Similarly, expression of *SPR* RNAi in IR76b neurons fully abolished the taste-dependent egg-laying preference behavior of mated females (Figs 2C and S1C). We then refined the experiment with another, significantly more specific Gal4 driver, IR41a-Gal4, targeting only the small number of olfactory neurons sensing polyamine odor (*IR41a-Gal4;UAS-SPRi*). We observed a similar reduction in attraction to polyamine odor in the T-maze compared to knockdown with IR76b-Gal4 in mated females (Fig 2D). By contrast, egg-laying preference was comparable to control mated females (Figs 2E and S1D). This result was consistent with the absence of IR41a-Gal4 expression in IR76b gustatory neurons [33]. These data were consistent with the hypothesis that SPR in chemosensory neurons is necessary to modulate the attraction of females to the smell and taste of polyamines after mating.

Given the central role of SPR in the classical post-mating switch, we asked whether SPR in chemosensory neurons was not only necessary but also sufficient to modulate their sensitivity. To this end, we re-expressed SPR in *SPR* mutant females in all IR76b neurons (IR76b-Gal4, polyamine taste and olfaction), in bitter taste neurons (GR66a-Gal4), or just in the olfactory subset of IR76b-expressing neurons that express IR41a (IR41a-Gal4) and assayed olfactory behavior (T-maze) and taste-dependent oviposition behavior. We found that re-expression of SPR in IR76b neurons fully rescued the *SPR* mutant phenotype of mated females in olfaction as well as in oviposition behavior (Fig 2F and 2G). Expression of SPR in GR66a bitter neurons, by contrast, had no effect on the *SPR* mutant phenotype in either of the two choice behaviors (Fig 2F and 2G). Re-expression of SPR selectively in IR41a OSNs did not rescue oviposition behavior of *SPR* mutant females, consistent with the fact that the egg-laying choice is mediated by taste neurons (Fig 2G). It did, however, rescue the olfactory attraction of *SPR* mutant females to polyamine odor in the T-maze (Fig 2F). This suggests that SPR plays a cell-autonomous role in a specific set of peripheral chemosensory neurons independent of its function in the cells in the female reproductive system.

Altogether, based on these data, we propose that SPR regulates choice behavior in a mating-state-dependent manner directly in chemosensory neurons, providing a mechanistic link between mating state and the neurons that process odors and taste.

### Mating and SPR Signaling Enhance Sensitivity of Gustatory Neurons

SPR signaling in chemosensory neurons appears to be required for the change in choice behavior after mating. This genetic mechanism could influence neuronal physiology at several levels of olfactory and taste processing starting at the peripheral level.

We have previously shown that IR76b taste neurons on the labellum are of particular importance for egg-laying choices on polyamine substrates [33]. Loss of IR76b completely abolishes the egg-laying preference of a mated female [33].

**Fig 3:**
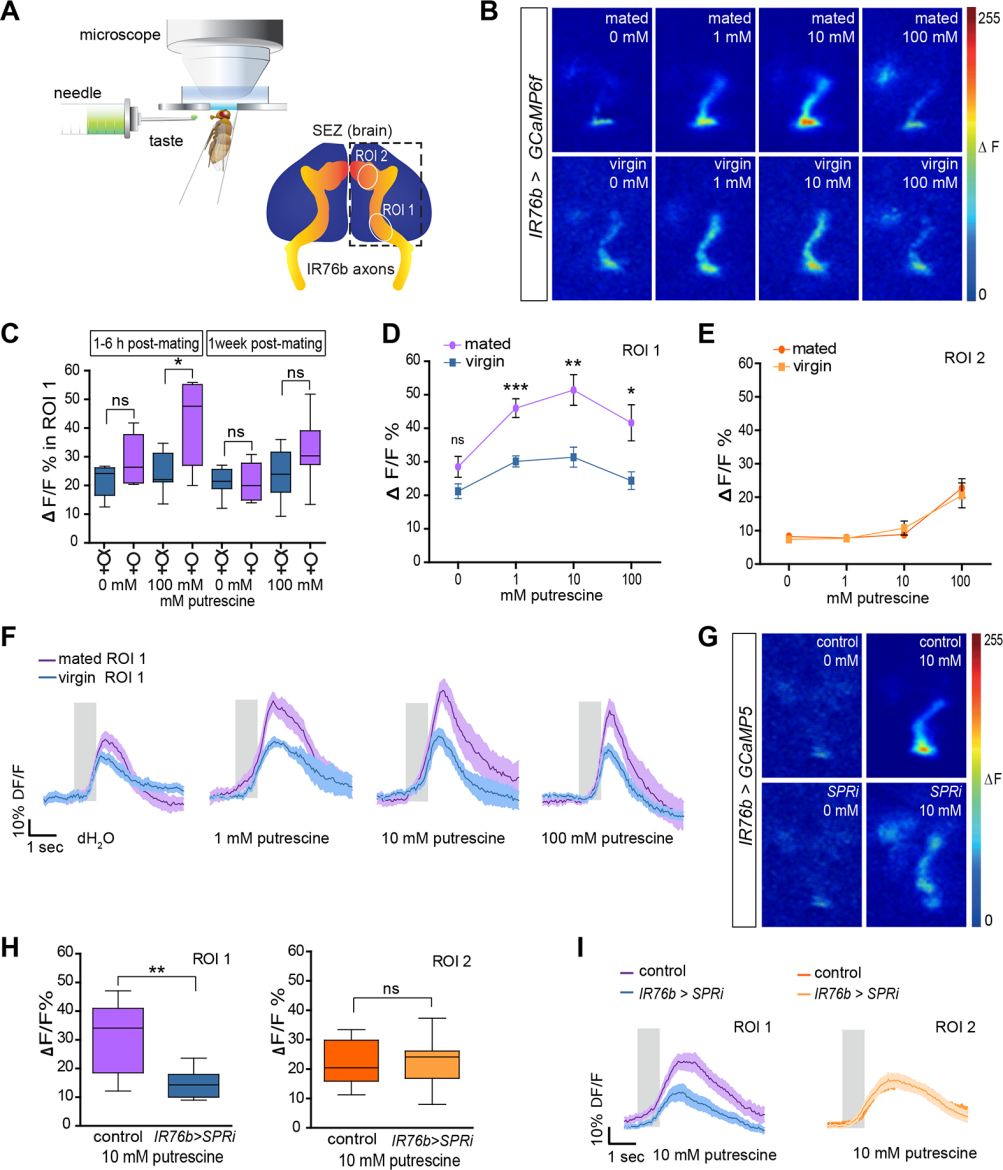
Mating increases sensitivity of taste neurons through SPR. (A) Scheme of the SEZ in vivo calcium imaging setup (top). Illustration of the SEZ area showing the innervation pattern of IR76b taste neuron axons (bottom). ROI 1 and ROI 2 delineate the regions of interest (ROI) used for quantification of the relative change in GCaMP-fluorescence (%ΔF/F). (B) Representative images of SEZ imaging of *IR76b-Gal4; UAS-GCaMP6f* mated and virgin female flies stimulated with distilled water (0 mM), 1 mM putrescine (1 mM), 10 mM putrescine (10 mM), and 100 mM putrescine (100 mM), respectively. (C) IR76b taste neuron terminals of mated females show a significantly increased response to putrescine after mating. While the response is highly significant at 1–6 h post-mating, it remains only a trend at 1 wk post-mating (*n* = 7). (D) Females at 1–6 h post-mating show higher IR76b taste neuron responses. GCaMP6f-fluorescence peak responses were quantified (in %ΔF/F) in the ROI 1 area. Flies were stimulated with increasing concentrations of putrescine (*n* = 7). (E) IR76b taste neurons of the same females as in (D) show no difference in the ROI 2 area. (F) Average response trace of the ROI 1 area (*n* = 7). The gray bar illustrates the stimulation period. The dark colored line in the middle presents the average value, and the light shade presents the SEM. (G) Representative images of IR76b GRN axons in the SEZ of test (*IR76b-Gal4*,*UAS-SPRi;UAS-GCaMP5*) and control (*IR76b-Gal4;UAS-GCaMP5*) females at 1–6 h post-mating. Flies were stimulated with distilled water (0 mM) and 10 mM putrescine (10 mM). (H) Quantification of peak responses (in %ΔF/F) of IR76b axon terminals of *IR76b-Gal4*,*UAS-SPRi;UAS-GCaMP5* and control (*IR76b-Gal4;UAS-GCaMP5*) females at 1–6 h post-mating (*n* = 8). Box plots show median and upper/lower quartiles, and whiskers show minimum/maximum values. (I) Average response trace of ROI 1 and ROI 2 area of IR76b axons in the SEZ of *IR76b>SPRi* and control females (*n* = 8). All *p*-values were calculated using an unpaired t-test (*p ≤ 0.05, **p ≤ 0.01, ***p ≤ 0.001).

To test whether mating modulates the sensitivity of gustatory neurons, we examined the activity of IR76b chemosensory neurons by recording their Ca^2+^ responses to polyamines at the level of their axon terminals in the SEZ of the central brain (Fig 3A). Because mating induces short-term (<24 h) and long-term (~1 wk) effects [46,47], we performed these experiments at two different time points: at 1–6 h or at 1 wk post-mating (Fig 3B–3F). We measured Ca^2+^ increases by recording GCaMP6f signals in IR76b axon terminals in the SEZ (*IR76b-Gal4;UAS-GCaMP6f*), which we divided based on the innervation pattern of IR76b neuron subsets into two broader innervation zones, region of interest (ROI) 1 and ROI 2 (Fig 3A). At 1–6 h post-mating, labellar IR76b neurons projecting to ROI 1, the primary response area for polyamines [33], responded significantly more strongly to a putrescine taste solution in mated females than in virgin females (Fig 3C and 3D). Interestingly, this was not the case for ROI 2, which responded significantly only to higher concentrations of putrescine (10–100 mM). IR76b neurons projecting to this region of the SEZ of virgin and mated females showed a similar response (Fig 3E). Interestingly, at the later time point (1 wk post-mating), the difference observed for axons projecting to ROI 1 was no longer significant. Hence, we conclude that mating transiently increases the sensitivity of polyamine-detecting IR76b labellar taste neurons after mating.

Is this shift of sensitivity in the GRNs mediated by SPR signaling directly in chemosensory neurons as the behavioral data suggests? To answer this, we recorded GCaMP signals from polyamine-sensitive taste neurons of mated females, in which we triggered RNAi against *SPR*. Knock-down of *SPR* in IR76b GRNs (*IR76b-Gal4*,*UAS-GCaMP5;UAS-SPRi*) of mated females led to a significant decrease in the presynaptic calcium increase of these neurons in response to polyamine taste compared to the response of mated controls (Fig 3G–3I). Notably, *SPR* knockdown had no effect on the response of IR76b neurons projecting to the ROI 2 region of the SEZ. These neurons responded like control neurons (Fig 3H and 3I), suggesting that SPR modulation only occurred in neurons that were affected by the mating state.

These results provide a mechanistic explanation for behavioral change occurring in the oviposition choice behavior of females upon mating, and they are consistent with our model that SPR in GRNs directly modulates sensory neuron sensitivity and thereby regulates choice behavior.

### Mating and SPR Signaling Decreases Responsiveness of Olfactory Neurons to Polyamines

**Fig 4:**
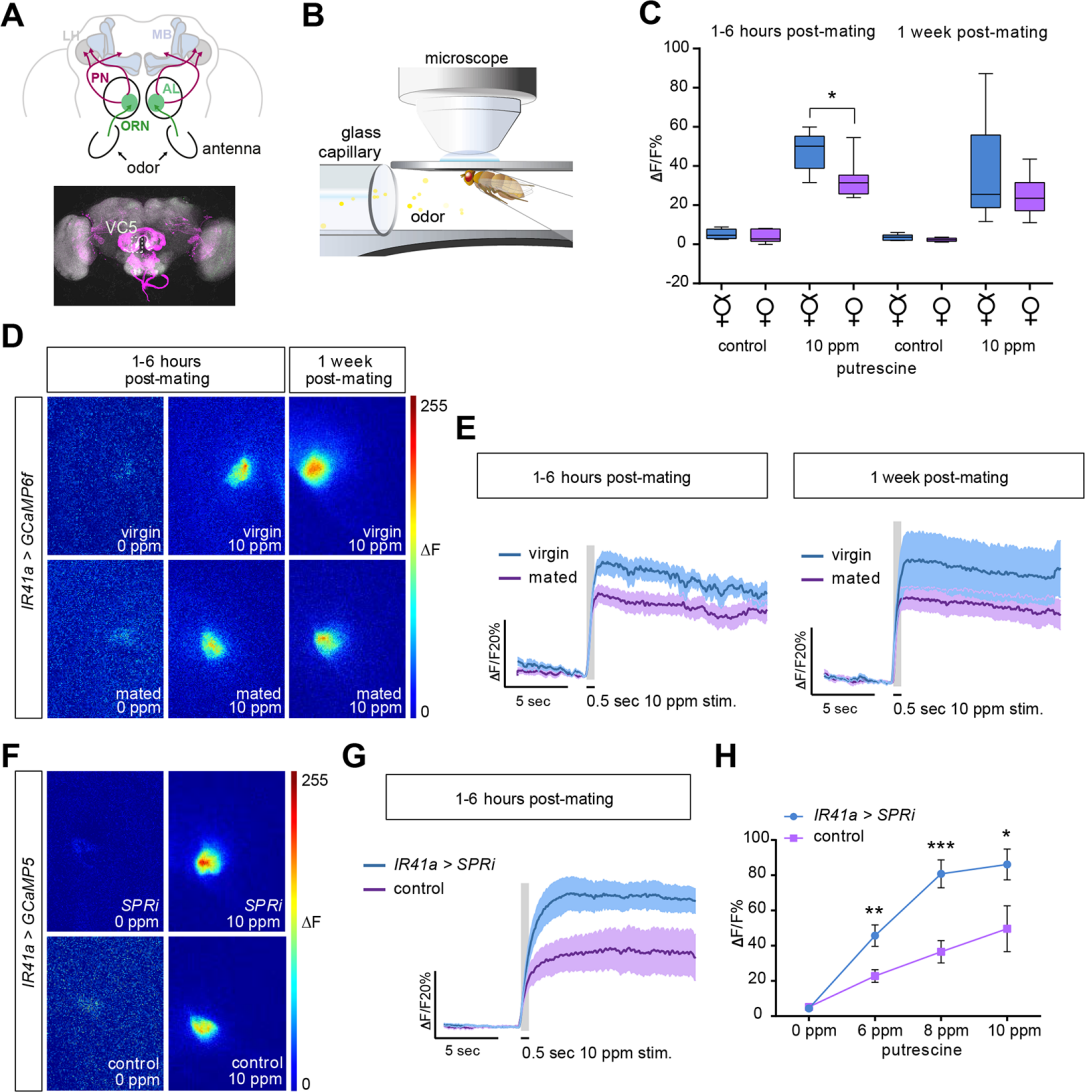
SPR decreases sensitivity of olfactory neurons to polyamines after mating. (A) Schematic diagram of a fly brain and its antennal appendages with olfactory sensory neurons (OSNs). OSNs project into the antennal lobe (AL), where they innervate a specific glomerulus (green). Projection neurons (PN) send the information mainly to two higher brain centers, the mushroom body (MB) and the lateral horn (LH) (top). Illustrative confocal image stack showing the IR41a and IR76b OSN innervation in the AL (bottom). VC5 is the glomerulus innervated by the polyamine-responding IR41a/IR76b sensory neurons. (B) Illustration of the in vivo calcium imaging setup. (C–E) In vivo calcium imaging of *IR41-Gal4;UAS-GCaMP6f* flies stimulated with water and 10 ppm putrescine, respectively. Mated females’ OSN axon terminals show a significant reduction in their sensitivity to putrescine at 1–6 h post-mating. (C) Quantification of peak ΔF responses (in %ΔF/F) in virgin and mated females. Boxes show median and upper/lower quartiles, and whiskers show minimum/maximum values. *p < 0.05, unpaired *t* test (*n* = 8). (D) Representative pseudo-color images showing the response to water and 10 ppm putrescine in virgin and mated flies at 1–6 h and 1 wk post-mating. (E) Average response trace (in %ΔF/F) of the VC5 glomerulus peak response at 1–6 h and 1 wk post-mating compared to traces from virgin females. The dark colored line in the middle presents the average value and the light shade presents the SEM. (F–H) In vivo calcium imaging of test (*IR41a-Gal4*,*UAS-SPRi;UAS-GCaMP5*) and control (*IR41a-Gal4;UAS-GCaMP5)* mated female flies. OSN axon terminals of *IR41a>SPRi* females show significantly enhanced responses to putrescine compared to control females. (F) Representative pseudo-color images showing the response to water and 10 ppm putrescine in *IR41a>SPRi* and control females, respectively. (G) Average response trace of the VC5 glomerulus in *IR41a>SPRi* and control females at 1–6 h post-mating for 10 ppm putrescine. (E,G) The gray column represents the 0.5 s stimulation period. Dark colored line is the average response and the light shade is the SEM. (H) Quantification of peak ΔF responses (in %ΔF/F) in *IR41a>SPRi* and control females for 0 ppm, 6 ppm, 8 ppm, and 10 ppm putrescine, respectively (*n* = 7 ± SEM). All *p*-values were calculated using an unpaired *t* test (*p ≤ 0.05, **p ≤ 0.01, ***p ≤ 0.001).

Olfactory preference behavior appears to undergo a similar shift as gustatory preference behavior after mating. We therefore carried out a set of experiments in the olfactory system similar to those described above. Axons of OSNs project centrally to the AL, the functional equivalent of the vertebrate olfactory bulb. This first-order olfactory information is further processed by local interneurons and then transferred by projection neurons (PNs) to higher brain centers (Fig 4A) [48]. Recent studies have shown that hunger enhances olfactory sensitivity to food odor by increasing presynaptic responses of OSNs via the OSN-resident short neuropeptide F (sNPF) and its receptor, sNPFR [42,43]. Metabolic state thereby regulates the efficacy of the synapse between OSN and PN similarly to what we have observed for mating state and GRNs.

To test whether OSNs are modulated in a similar manner as GRNs, we imaged calcium increases of IR41a axon terminals at the level of the AL (*IR41a-Gal4;UAS-GCaMP6f*) (Fig 4B). Surprisingly, we observed that mating significantly decreased the response of these neurons to behaviorally relevant concentrations of polyamines (Fig 4C–4E). As in the gustatory system, this decrease was strongly significant at 1–6 h and remained only a trend at 1 wk post-mating (Fig 4C). In contrast to GRNs, however, mating transiently suppresses the sensitivity of OSNs. How does this result explain the behavioral shift toward higher polyamine levels after mating? Virgins show highest attraction to very low levels of polyamines and reduced attraction or enhanced aversion at levels preferred by mated females (Fig 1B). By contrast, mated females show the highest attraction to relatively high amounts of polyamine, which roughly corresponds to decaying fruit (10 ppm/1 mM; Fig 1B). It was previously shown that different odor concentrations can have differential behavioral effects and can even recruit different PNs downstream of the same OSNs [49,50]. Such a mechanism could also explain the change of behavior to polyamines, whereby a reduction of olfactory sensitivity may change higher olfactory processing and consequently shift the mated female’s preference to increased levels of beneficial polyamines for egg-laying.

Again, we asked whether this change in sensitivity was mediated by SPR signaling in OSNs themselves, as the behavioral data would suggest. As in the gustatory system, this appeared to be the case for the olfactory system, as knockdown of *SPR* in IR41a OSNs resulted in a significant change in presynaptic calcium responses of these neurons (Fig 4F–4H). As predicted from the comparison of mated and virgin OSN responses to putrescine, we observed a greater increase of GCaMP fluorescence in OSN axon terminals of mated females with *SPR* knockdown (*IR41a-Gal4*,*UAS-GCaMP5;UAS-SPRi*) compared to mated genetic controls (Fig 4H).

Together, we interpret these data to mean that SPR in chemosensory neurons regulates the sensitivity of OSNs and GRNs to polyamines directly at the level of these chemosensory neurons. This change in sensitivity follows two different neural mechanisms, i.e., increased calcium responses of GRN and decreased responses of OSN axon terminals. This, in turn, appears to alter the mated female’s perception and adjusts her choice behavior to polyamines.

### Myoinhibitory Peptides Regulate Polyamine Sensitivity in the Context of Mating

**Fig 5:**
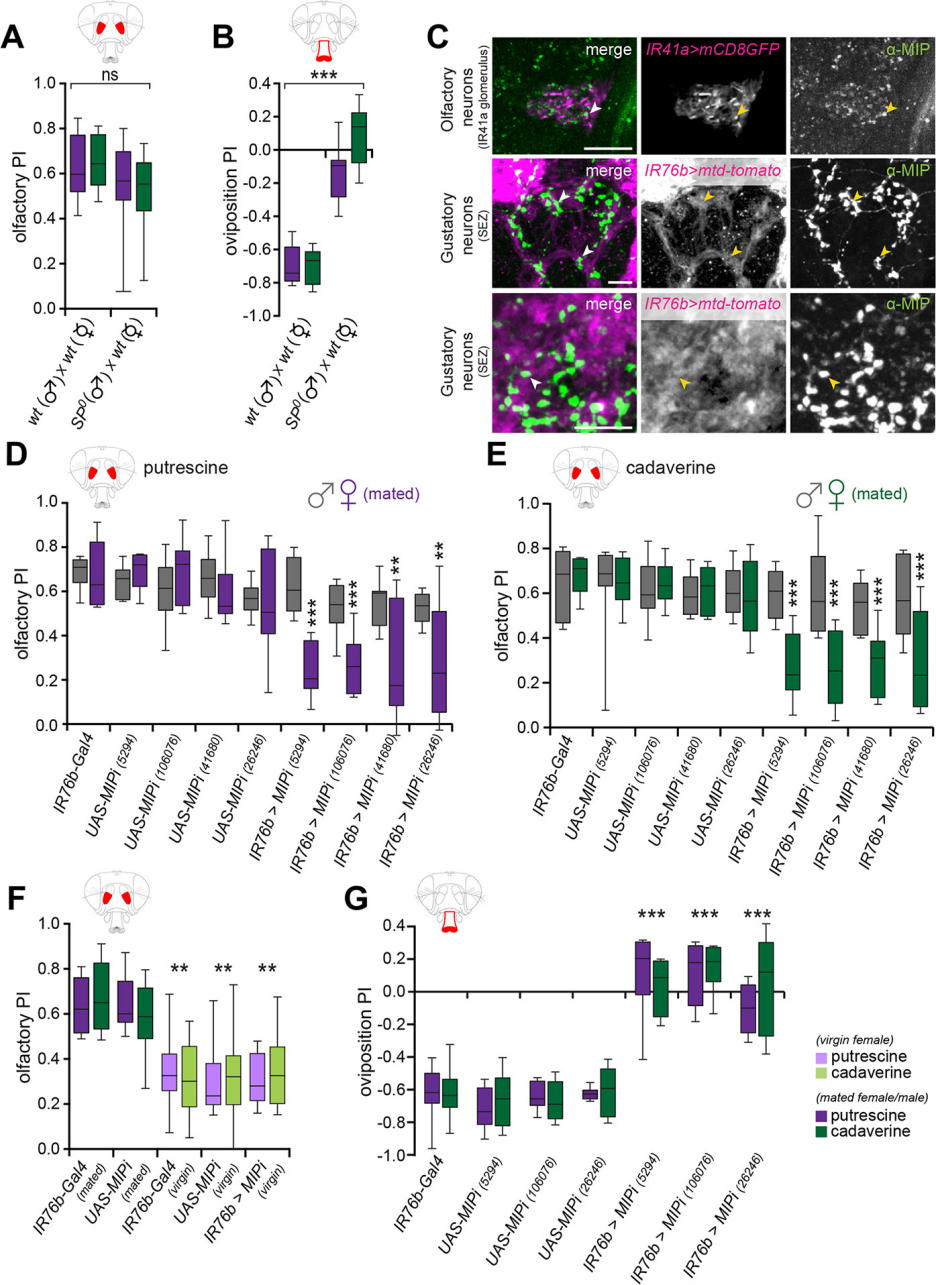
Myoinhibitory peptides regulate polyamine sensitivity in the context of mating. (A) Loss of sex peptide (SP) in the sperm of the male does not significantly affect chemosensory attraction of mated females to 10 ppm of polyamines. Wild-type (wt) Canton S females mated to wild-type or sex peptide mutant (*SP*^*0*^) males do not show a significantly altered level of attraction to the odor of putrescine or cadaverine. (*n* = 8, 60 flies/trial). (B) *SP*^*0*^ male-mated Canton S females lay their low numbers of eggs on either site of the oviposition assay and show no preference behavior. (*n* = 8, 60 flies/trial). (C) Myoinhibitory peptide (MIP) expression in the AL and SEZ regions in the female brain. In the AL, the glomerulus innervated by IR41a OSNs is displayed (*IR41a-Gal4;UAS-mCD8GFP*). Note that MIP staining is detected in close proximity to IR41a axon terminals. In the SEZ, anti-MIP staining (green) localizes close to IR76b neuron axons and axon terminals (magenta) consistent with MIPs being secreted by IR76b neurons (*IR76b-QF;QUAS-mtd-tomato*) (see arrowheads). (D,E) MIPs modulate olfactory attraction to polyamines selectively in mated females but not males. RNAi-mediated knockdown of MIPs with four different RNAi transgenic lines in IR76b neurons (*IR76b-Gal4;UAS-MIPi*) selectively reduces the olfactory preference of mated females but not of males to 10 ppm of putrescine (D) or 10 ppm of cadaverine (E) (*n* = 8, 60 flies/trial). (F) The effect of MIP knockdown (*IR76b-Gal4;UAS-MIPi*) depends on the mating state of the female, as the low attraction of virgin females to 10 ppm polyamine odor was not further reduced in virgin females with MIP knockdown compared to virgin controls without RNAi against MIPs. Box plots show median and upper/lower quartiles (*n* = 8, 60 flies/trial). (G) Knockdown of MIPs in IR76b neurons abolishes oviposition preference to 1 mM putrescine and cadaverine using three different MIPi transgenic lines (*IR76b-Gal4;UAS-MIPi*). Females laid their eggs on either side of the assay. All box plots show median and upper/lower quartiles (*n* = 8, 60 flies/trial). All *p*-values were calculated via two-way ANOVA with the Bonferroni multiple comparison post-hoc test (ns > 0.05, *p ≤ 0.05, **p ≤ 0.01, ***p ≤ 0.001).

We showed that polyamine perception changes upon mating and that this change is mediated by SPR signaling in chemosensory neurons. How SPR signaling is triggered in chemosensory neurons, however, remains unclear. The best-characterized SPR ligand is SP itself. A role for SP in feeding behavior was demonstrated previously. For instance, SP provided by the male stimulates feeding in mated females, and *SP* mutant male-mated females do not show this increase [10]. Furthermore, the mated female’s feeding preference for yeast and salt depends on SP provided by the male during mating [9,41]. Here, SP activates the canonical SPR pathway through *ppk*-positive SPR neurons in the female’s oviduct, which leads to a change in feeding preference. Whether and how mating and/or SP alter the sensitivity of taste neurons to yeast or salt or their higher-order chemosensory processing is not known. Furthermore, in the present context, if SP were to act directly on the chemosensory neurons, some SP would have to be transferred from its point of delivery, the female reproductive tract, to SPR in chemosensory neurons on the head. To test the requirement of SP in the sensitivity to polyamines, we crossed males that were mutant for the *SP* gene (*SP*^*0*^), and thus lacking SP from their semen, to wild-type virgin females [51]. We compared the behavior of these females to that of females mated to wild-type males. Interestingly, the attraction of females mated to *SP*^*0*^ males to polyamine odor in the T-maze was not significantly different from females mated to wild type males (Fig 5A). This suggested that SP was not the key to mating-state-dependent olfactory sensitivity modulation. Furthermore, it also indicated that changes in feeding behavior as reported by Carvalho et al. [10] are not necessary for the observed olfactory modulation. We also analyzed the contribution of SP to oviposition preference. *SP*^*0*^ mated females appeared to show the same lack of preference as virgin flies and laid their very few eggs on either side of the assay (Fig 5B). Nevertheless, the olfactory preference data as well as the site of action of SP indicated that another additional ligand was involved in mating-state-dependent chemosensory changes in females. Moreover, this result was in agreement with our data showing that re-expression of SPR in gustatory or olfactory neurons was sufficient to modulate their responses to polyamines.

We therefore asked whether MIPs could be the functional ligands of SPR at the level of the chemosensory neuron central projections and could mediate the modulation of polyamine behavior. The expression of MIPs in the vicinity of IR41a axon terminals in the AL and in the vicinity of IR76b axons and axon terminals in the SEZ (Fig 5C) is consistent with their possible requirement in the chemosensory neurons themselves. We employed four different, independent RNAi-triggering transgenic lines to knockdown the expression of MIPs in IR76b-positive sensory neurons and tested fly behavior in the T-maze (olfaction) and oviposition (taste) assays. RNAi-mediated suppression of MIP expression in chemosensory neurons (*IR76b-Gal4;UAS-MIPi*) reduced the expression of MIP in chemosensory processing centers, but not in the rest of the brain as compared to controls or knockdown with a pan-neural driver (S5A Fig). Importantly, this manipulation (*IR76b-Gal4;UAS-MIPi*) also significantly lowered the attraction of mated females to polyamines in the T-maze as compared to genetic controls (Fig 5D and 5E). Notably, although MIP expression appears highly similar between males and females (S4 Fig) [52], this reduced olfactory attraction was only observed in females, but not in male flies (Fig 5D and 5E). These data mirror the lack of olfactory phenotype in the *SPR* mutant male (see Fig 1G) and further supported our model of a gender-specific role for SPR signaling. Furthermore, similar to what was observed upon *SPR* knockdown (see Fig 2C), virgin female attraction to polyamines was not further decreased when MIPs were down-regulated by RNAi, showing that the effect of MIP was mating-state-dependent (Fig 5F).

Finally, a similar analysis in the context of oviposition behavior showed that knockdown of MIPs in IR76b neurons (*IR76b-Gal4;UAS-MIPi*) had the same effect on female oviposition behavior as knockdown of *SPR* (Fig 5G). Female flies laid their eggs in equal numbers on polyamine-rich and control substrates (S5B Fig).

These data describe a role for MIPs in female reproductive behavior and indicate that they regulate polyamine-mediated chemosensory behavior presumably as ligands for SPR. Furthermore, similar to sNPF and its receptor [43], MIPs and SPR appear to be required directly in gustatory and olfactory neurons. In contrast to sNPF and sNPFR, SPR and MIPs are only required in the female.

### Interaction of Mating State and SPR/MIP Signaling

**Fig 6:**
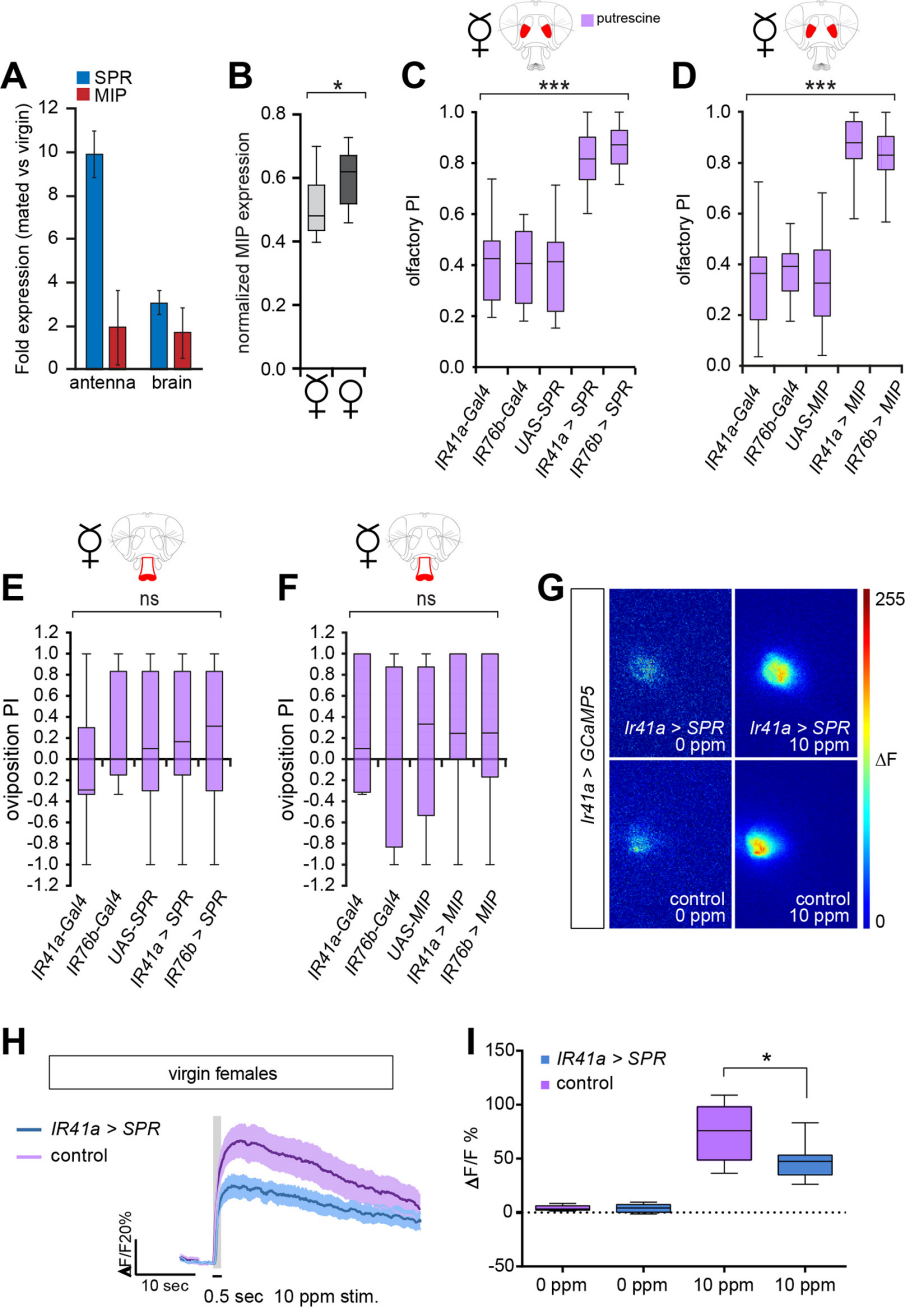
MIP expression is increased in the AL upon mating. (A) SPR and MIP expression analysis before and after mating of antenna and brain of virgin or mated females. Quantitative PCR (*n* = 3 genetic variants with 200 females per *n* and condition) of the antenna and brain of virgin and mated flies reveals that SPR expression upon mating increases upon mating ~10-fold in the antenna and ~3-fold in the brain. Graph displays 2^∆∆CT ± SEM (see Materials and Methods for details). (B) Quantification of MIP protein expression in the AL. Mated flies show a small but significant increase of MIP expression in the AL. *n* = 20 flies per group. *p = 0.0113, unpaired *t* test. (C) Overexpression of SPR under the control of the IR41a enhancer (*IR41a-Gal4;UAS-SPR*) or IR76b enhancer (*IR76b-Gal4;UAS-SPR*) in virgin females increases their attraction to polyamine odor in the T-maze assay (*n* = 8). (D) Overexpression of MIP under the control of the IR41a enhancer (*IR41a-Gal4;UAS-MIP*) or IR76b enhancer (*IR76b-Gal4;UAS-MIP*) in virgin females induces a strongly increased attraction to polyamine odor in olfactory T-maze assay (*n* = 8). (E,F) No egg-laying preference was observed in virgin females overexpressing SPR or MIP under the control of the IR41a enhancer (*IR41a-Gal4;UAS-SPR* or *IR41a-Gal4;UAS-MIP*) or IR76b enhancer (*IR76b-Gal4;UAS-SPR* or *IR76b-Gal4;UAS-MIP*) in oviposition assays compared to controls (*n* = 8). Virgin females overexpressing SPR or MIP laid very few eggs, similar to control virgins, which results in the high variability observed in the data. (G–I) In vivo calcium imaging of presynaptic terminals of OSNs in the AL expressing *IR41a-Gal4*,*UAS-SPR;UAS-GCaMP5* or *IR41a-Gal4;UAS-GCaMP5* (control). Virgin females overexpressing SPR in IR41a OSNs show significantly suppressed calcium signals to putrescine compared to virgin control females. (G) Representative pseudo-color images showing the response to 0 ppm and 10 ppm putrescine in SPR-overexpressing and control virgin females, respectively. (H) Average activity trace of the VC5 glomerulus in SPR-overexpressing and control virgin females for 10 ppm putrescine. (I) Quantification of peak ΔF responses in SPR-overexpressing (*n* = 7) and control (*n* = 8) females for 0 ppm and 10 ppm putrescine. Boxes show median and upper/lower quartiles, and whiskers show minimum/maximum values. All *p*-values were calculated via two-way ANOVA with the Bonferroni multiple comparison post-hoc test (ns > 0.05, *p ≤ 0.05, **p ≤ 0.01, ***p ≤ 0.001) except for Fig 6B and 6I, where *p*-values were calculated via an unpaired t-test (*p ≤ 0.05, **p ≤ 0.01, ***p ≤ 0.001).

Mating appears to induce a change in SPR signaling not only in the female reproductive tract as previously shown [15], but also in her chemosensory neurons. In the female reproductive tract, SP is only available upon mating. How is this change brought about in peripheral neurons or in regions of the central brain such as the AL or SEZ? The most straightforward mechanism would be an alteration in the expression of MIP or SPR upon mating such that there is more functional SPR or available MIP in the mated female compared to the virgin. Notably, MIP expression cycles with the circadian rhythm of the fly, in line with the role of SPR and MIP in maintaining a sleep-like state in flies [25]. As the authors did not observe any change in MIP mRNA levels, a post-transcriptional regulatory mechanism could be involved [25]. To test whether SPR and MIP expression was being modulated, we used quantitative PCR to compare mRNA levels of SPR and MIP before and after mating (Fig 6A). To this end, we dissected antennae and brains of virgins and mated females at 1–6 h after mating and compared the expression of MIP and SPR to a control mRNA not expected to change upon mating (see Materials and Methods). We found that SPR expression increases about 10-fold upon mating in the antenna but to a lesser extend in the brain (~3-fold, Fig 6A). By contrast, MIP expression after mating remained more similar to the expression before mating in both antenna and brain (Fig 6A). These data are consistent with our hypothesis that SPR expression is selectively increased in chemosensory neurons upon mating and modulates female preference behavior. Furthermore, it strengthens the conclusion reached by genetic experiments that SPR signaling is required in chemosensory neurons.

While we were not able to challenge or confirm this result by using antibody staining against SPR, both a previously published antibody [15] and another antibody that we produced ourselves showed similar stainings in wild-type and *SPR* mutant brains (S6A–S6C Fig), we sought to quantify MIP protein expression at the level of the OSN terminals in the AL. This was especially important because MIP expression was previously suggested to be regulated at the level of the protein and not at the level of the mRNA [25]. Antibody staining against MIPs reveals central neurons as well as axon tracts of peripheral neurons projecting into the brain (Figs 5C and S4). In the SEZ, passing neuronal tracts of central neurons dominate (S4 Fig) and unfortunately mask the MIP-stained axons projecting from peripheral taste organs, including the proboscis (Figs 5C and S4; see arrowheads). This situation prevented us from quantifying MIP expression selectively in GRNs. In the olfactory system, nevertheless, MIP expression was defined and appeared to stem only from OSNs and from local interneurons. Although MIP protein expression analysis did not show any gross differences between mated and virgin females (S4 Fig), using more detailed image quantification we observed a significant increase of MIP expression in the AL in mated compared to virgin females (Figs 6B and S7A). While this increase appears small, it is statistically significant.

These results suggest that mating leads to a marked increase of SPR in chemosensory organs. This increase in SPR expression, accompanied by a small increase in MIP expression, might be the trigger for the mating-state-dependent modulation of polyamine taste and smell neurons. Of note, hunger modulates levels of the receptor sNPFR but not the expression of the neuropeptide itself [43].

Based on these results, we tested the effect of overexpression of SPR or MIP in chemosensory neurons in virgin females. We overexpressed SPR and MIP under the control of the IR76b enhancer (*IR76b-Gal4*) in all IR76b neurons (taste and olfaction) as well as only in OSNs under the control of the IR41a enhancer (*IR41a-Gal4*) in virgin females and tested their preference for polyamines. These manipulations had no effect on the number of eggs that virgin females laid, and egg numbers remained very low and similar to control virgins (S7B Fig). In contrast to the unchanged egg-laying activity, virgin females overexpressing SPR in chemosensory neurons showed a strongly increased attraction to polyamine odor (Fig 6C). This was true regardless of whether SPR was overexpressed under the control of IR76b-Gal4 or selectively in OSNs using IR41a-Gal4 (Fig 6C). We observed similar results in a reminiscent experiment, in which we overexpressed MIP instead of SPR. Also in this case, virgin females with increased levels of MIP in their chemosensory neurons showed a significantly increased preference for high polyamine levels compared to control virgins (Fig 6D). We also tested oviposition behavior. Given the low numbers of eggs, however, these data were less revealing and very variable, as small changes in egg-placing preference lead to large changes in preference index. In spite of these limitations, no clear preference was observable in virgins overexpressing SPR or MIP and control virgins (Fig 6E and 6F).

Our data would predict that the observed change in choice behavior upon overexpression of SPR is triggered by sensory neuron modulation. To analyze this, we used in vivo calcium imaging as described above. Indeed, we found that in virgins, overexpression of SPR selectively in IR41a OSNs significantly reduced the presynaptic response of IR41a neurons to polyamines compared to controls (Fig 6G–6I). This result was the exact opposite of the effect seen when SPR expression was knocked down using RNAi in IR41a OSNs (see Fig 3) and correlated well with the observed behavior of virgins overexpressing SPR.

In conclusion, expression analysis in conjunction with behavioral and imaging analysis leads us to propose that mating induces primarily an increase of SPR expression in chemosensory neurons. Boosted levels of SPR activated by mildly increased levels of MIPs modulate chemosensory neuron output in response to polyamines and thereby increase female preference for higher concentrations of polyamines. Thus, SPR/MIP signaling in chemosensory neurons seems not only necessary and sufficient but, as these data indicate, an instructive signal adjusting choice behavior to reproductive state.

## Discussion

**Fig 7:**
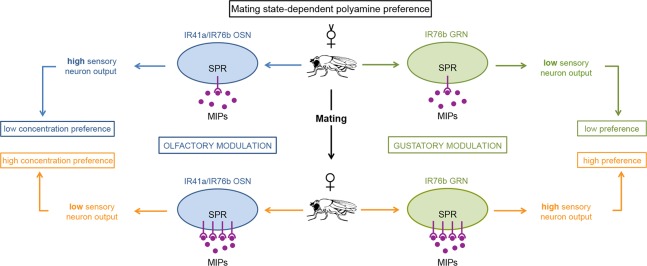
SPR/MIP signaling in chemosensory neurons adjusts female preference behavior upon mating. Model for mating-state-dependent modulation of olfactory and gustatory polyamine preference. (Left side) Upon mating, increased amounts of SPR in the sensory neuron suppress the output of IR41a/IR76b olfactory neurons, thereby increasing the female’s preference for higher concentrations of polyamine. (Right side) Mating increases the SPR amount in gustatory sensory neurons, and in contrast to the situation in OSNs increases the presynaptic output of IR76b taste neurons. This change increases the mated female’s preference for polyamine taste. In conclusion, mating increases SPR expression in chemosensory neurons and by two different cellular mechanisms enhances the mated female’s preference for beneficial polyamines.

Here, we describe a novel mechanism that enhances the sensitivity of chemosensory neurons to match the choice behavior of a gravid female to her increased nutritional needs. Female *Drosophila* use polyamines to identify and evaluate beneficial food and egg-laying sites with specific olfactory and taste receptor neurons. We demonstrate that this multisensory detection of polyamines undergoes reproductive state-dependent peptidergic modulation. Mechanistically, we show that virgin females, or mated females lacking the G-protein coupled receptor SPR, display reduced preference for polyamine-rich food and oviposition sites. Using targeted gene knockdown, mutant rescue, overexpression, and in vivo calcium imaging, we thus unravel a new role for SPR and its conserved ligands, MIPs, in directly regulating the sensitivity of chemosensory neurons and modulating taste and odor preferences according to reproductive state (Fig 7). Together with recent work in the mouse [6], our results emphasize that chemosensory neurons are potent targets for tuning choice behavior to reproductive state.

### Neuropeptidergic Modulation of Chemosensory Neurons Regulates Mating State-Dependent Choice Behavior

Reproductive behaviors such as male courtship and female egg-laying strongly depend on the mating state [8,9,29,31,53]. While previous work has suggested that mating modulates odor- or taste-driven choice behavior of *Drosophila* females [9,29,31,41,54], how mating changes the processing of odors and tastes remained elusive. We show here that a female-specific neuropeptidergic mechanism acts in peripheral chemosensory neurons to enhance female preference for essential nutrients. Our data suggests that this modulation is autocrine and involves the GPCR SPR and its conserved MIP ligands. Notably, MIPs are expressed in chemosensory cells in the apical organs of a distant organism, the annelid (*Platynereis*) larvae, in which they trigger settlement behavior via an SPR-dependent signaling cascade [22]. Importantly, as SP and not MIP induces the SPR-dependent canonical post-mating switch [15,19], our findings report the first gender and mating-state-dependent role of these peptides [25]. Whether this regulation is also responsible for previously reported changes in preference behavior upon mating [9,29,31,41] remains to be seen, but we anticipate that this type of regulation is not only specific to polyamines. On the other hand, mating-dependent changes for salt preference—salt preference is also dependent on IR76b receptor but in another GRN type—might undergo a different type of regulation, as RNAi-mediated knockdown of SPR in salt receptor neurons had no effect on salt feeding [41]. Instead, the change in salt preference is mediated by the canonical SP/SPR pathway and primarily reflects the fact that mating has taken place. The mechanism of how salt detection and/or processing are modulated is not known. In contrast to salt preference and polyamine preference, acetic acid preference is strongly modulated by egg-laying activity and not just mating [31]. The extent to which changes in salt or acetic acid preference are similar to the modulation of behavior to polyamine that we describe here can currently not be tested, because the olfactory neurons that mediate acetic acid preference have not been determined [31].

### G-protein Coupled Receptor Signaling Has the Opposite Effect on Olfactory versus Gustatory Neurons

While SPR regulates the neuronal output of both olfactory and gustatory neurons, our behavioral and our physiological data surprisingly revealed that it does so through two opposite neuronal mechanisms. SPR signaling increases the presynaptic response of GRNs and decreases it in OSNs. Behaviorally, these two types of modulation produce the same effect: they enhance the female’s attraction to polyamine and tune it to levels typical for decaying or fermenting fruit. How these two effects are regulated by the same receptor and ligand pair remains open. GPCRs can recruit and activate different G-proteins. SPR was previously shown to recruit the inhibitory Gα_i/o_-type, thereby down-regulating cAMP levels in the cell [19,55]. In the female reproductive tract, SP inhibits SPR-expressing internal sensory neurons and thereby promotes several post-mating behaviors [15]. This type of inhibitory G-protein signaling could also explain our data in the olfactory system. Here, mating decreases the presynaptic activity of polyamine-detecting OSNs, and conversely, RNAi knockdown of *SPR* increases their responses strongly. This decrease in neuronal output also shifts the behavioral preference from low to high polyamine levels. While the relationship between behavior and GRN activity is much more straightforward in the gustatory system (increased neuronal response, increased preference behavior), it implies that another G-protein might be activated downstream of SPR. G-protein Gα_i/s_ increases cAMP levels and Gα_q_ enhances phospholipase C (PLC) and calcium signaling [56]. In addition, Gβγ subunits regulate ion channels and other signaling effectors, including PLC [56]. Future work will address the exact mechanisms of this bi-directional modulation through SPR signaling. Nonetheless, it is interesting to speculate that different cells, including sensory neurons, could be modulated differentially by the same molecules depending on cell-specific states and the availability of signaling partners.

### Modulation of Polyamine Perception and Its Relationship to Reproductive State

While our data provides a neuronal and molecular mechanism of how chemosensory processing itself is affected by mating, it remains unclear how mating regulates MIP/SPR signaling in chemosensory neurons. Our data indicates that SPR levels strongly increase in chemosensory organs upon mating. In addition, MIP levels appear to be mildly increased by mating. This suggests that mating regulates primarily the expression of the GPCR resembling the modulation of sNPFR expression during hunger states. On the other hand, MIP overexpression also induced mated-like preference behavior in virgin flies, suggesting a somewhat more complex situation. For instance, it is possible that overexpression of MIP induces the expression of SPR. Alternatively, active MIP levels might also be regulated at the level of secretion or posttranslational processing, and overexpression might override this form of regulation. In the case of hunger, sNPFR levels are increased through a reduction of insulin signaling [43]. SP could be viewed as the possible equivalent of insulin for mating state. Females mated to *SP* mutant males, however, do not show a significant change in olfactory perception of polyamines. It is yet important to note that male sperm contains roughly 200 different proteins, some of which might be involved in mediating the change in MIPs/SPR signaling upon mating [7]. In the mosquito, which does not possess SP, the steroid hormone 20E serves as the post-mating switch [57]. Interestingly, mating or treatment with 20E induces in particular the expression of the enzymes required for the synthesis of polyamines in the female spermatheca, a tissue involved in sperm storage and egg-laying [57]. Whether such a mechanism also exists in *Drosophila* is not known.

In addition to mating and signals transferred by mating, it is possible that egg-laying activity contributes to the regulation of MIPs/SPR signaling in chemosensory neurons through a mechanism that involves previously identified mechanosensory neurons of the female’s reproductive tract; such neurons may sense the presence of an egg to be laid [31]. Indeed, females that cease to lay eggs return to polyamine preferences as found before mating. On the other hand, *SP* mutant male-mated females and *ovoD1* sterile females still show enhanced attraction to polyamine odor, although they lay very few or no eggs. Conversely, knockdown of *SPR* in IR41a neurons reduced polyamine odor attraction but had a marginal effect on the number of eggs laid. We observed, nevertheless, somewhat reduced numbers of eggs laid upon inactivation of IR76b neurons. At this point, we can only speculate about possible reasons. Although IR76b receptor is not expressed in ppk-positive internal SPR neurons, we do find expression of IR76b-Gal4 in neurons innervating the rectum and possibly gut (data not shown). Hence, there might be an IR76b-mediated interplay between metabolism and nutrient uptake that influences egg-laying. However, females mated to SP-mutant males do not display an increase in feeding [10], indicating that preference for polyamines does not depend on the metabolic cost of egg-laying. This conclusion is strengthened by the data obtained with mated *ovoD1* sterile females, who show similar attraction to polyamines as compared to mated controls. Due to very few or no eggs laid by *SP* mutant male-mated females and *ovoD1* females, respectively, we cannot, however, fully exclude a contribution of egg-laying activity to taste-dependent oviposition choice behavior.

A further argument against an important role of egg-laying activity in our paradigm comes from the observation that the sensory modulation of OSNs and GRNs occurs rapidly after mating and is maintained only for a few hours. Similarly, SPR expression increases within the same time window shortly after mating. Egg-laying, however, continues for several days after this. In addition, overexpression of SPR was sufficient to switch virgin OSN calcium responses and female behavioral preferences to that of mated females without increasing the number of eggs laid. All in all, these data are more consistent with the hypothesis that mating and not egg-laying activity per se is the primary inducer of sensory modulation leading to the behavioral changes of females.

It remains that the exact signal triggered by mating that regulates odor and taste preference for polyamines through the here-identified mechanism needs to still be determined. Furthermore, the role of metabolic need and polyamine metabolism is not completely clear. This is similar to the situation found for increased salt preference after mating. While more salt is beneficial for egg-laying, sterile females still increase their preference for salt upon mating [41]. Regardless, in the case of polyamines, it is tempting to speculate that exogenous (by feeding) and endogenous (by enzymatic activity or expression) polyamine sources are regulated by reproductive state and together contribute to reach optimal levels for reproduction in the organism.

### Modulated Sensory Perception Leads to Lasting Behavioral Changes

Our results bear some similarities to recent work on the modulation of OSN sensitivity in hunger states [43]. sNPF/sNPFR signaling modulates the activity of OSNs in the hungry fly. MIPs/SPR might play a very similar role in the mated female. Overexpression of sNPFR in OSNs of fed flies was sufficient to trigger enhanced food search behavior [43]. Likewise, an increase in SPR signaling in taste or smell neurons converts virgin to mated female preference behavior. Therefore, different internal states appear to recruit similar mechanisms to tune fly behavior to internal state. Furthermore, such modulation of first order sensory neurons appears not only be conserved within a species, but also for regulation of reproductive state-dependent behavior across species. For instance, a recent study in female mice showed that progesterone-receptor signaling in OSNs modulates sensitivity and behavior to male pheromones according to the estrus cycle [6]. Also in this case, sensory modulation accounts in full for the switch in preference behavior. What is the biological significance of integrating internal state at the level of the sensory neuron? First, silencing of neurons in a state-dependent manner shields the brain from processing unnecessary information. As sensory information may not work as an on/off switch, it is possible that an early shift in neural pathway activation might reduce costly inhibitory activity to counteract activation once the sensory signal has been propagated. Second, another interesting possibility is that peripheral modulation might help to translate transient changes in internal state into longer-lasting behavioral changes that manifest in higher brain centers. This might be especially important in the case of female reproductive behaviors such as mate choice or caring for pups or babies. In contrast to hunger, which increases with time of starvation, the effect of mating decays slowly over time as the sperm stored in the female’s spermatheca is used up [58]. We have shown that the effect of mating on chemosensory neurons mediated by MIPs/SPR signaling is strong within the first 6 h after mating and remains a trend at 1 wk post-mating. However, it triggers a long-lasting behavioral switch, which is observed for over a week. Therefore, this transient modulation and altered sensitivity to polyamines could be encoded more permanently in the brain when the animal encounters the stimulus, for instance, in the context of an excellent place to lay her eggs. Because polyamine preference continues to be high for as long as stored sperm can fertilize the eggs, we speculate that this change in preference might be maintained by a memory mechanism in higher centers of chemosensory processing. Thus, short-term sensory enhancement not only increases perceived stimulus intensity, it may also help to associate a key sensation to a given reward or punishment. These chemosensory associations are of critical importance in parent–infant bonding in mammals, including humans, which form instantly after birth and last for months, years, or a lifetime [59].

## Materials and Methods

### Fly Rearing and Lines

*Drosophila melanogaster* stocks were raised on conventional cornmeal-agar medium at 25°C temperature and 60% humidity and a 12 h light:12 h dark cycle. Following fly lines were used to obtain experimental groups of flies in the different experiments:

1. Canton S
2. *w*^*1118*^
3. w*;;*UAS-Kir2*.*1*::*eGFP*
4. w*;P[IR41a-GAL4.*2474]attP40;TM2/TM6B*
5. w*; P[IR76b-GAL4.*916]226*.*8;TM2/TM6B*
6. w*;GR66a-Gal4/Cyo;TM2/TM6B
7. *Df(1)Exel6234* (*SPR* loss of function mutant); the mutation was verified using two different primer sets: Primer Pair-1: CCACCGTAATCTTGGCCCTTTTC, GTGGACCCCGAGTGGAAAATAAAAG; Primer Pair-2: AAGGGAGTCGGTTACTTGCG, TTCGTTCGGGGGATGTCAAG (see S6 Fig)
8. w*;UAS-mCD8GFP
9. w*;*UAS-GCaMP6f*
10. w*;;*UAS-GCaMP5*
11. For sex peptide mutant males: *SP*^*0*^*/TM3 Sb* flies were crossed *to Δ130/TM3 Sb* (gift from Mariana Wolfner)
12. Lines for RNAi knockdown of MIP: #26246 (*y1 v1; P[TRiP*.*JF02145]attP2)*, #41680 (y1 sc* v1; #P[TRiP.*HMS02244]attP2*), #106076 (*P[KK106116]VIE-260B*), #5294 (*w*^*1118*^*; P[GD2689]v5294*)
13. Line for RNAi knockdown of SPR: *#106804 VDRC P[KK103356]VIE-260B*
14. w*;P[IR76b-QF.*1*.*5]2*
15. *w*^*1118*^*;UAS-mCD8GFP*,*QUAS-mtd-tomato-3xHA*
16. *w*^*1118*^*;UAS-SPR/CyO* (gift from Barry Dickson)
17. *w*^*1118*^*;UAS-MIP* (gift from Doug Allan)
18. *ovoD1* (#1309) sterile females were obtained by crossing *ovoD1* males to Canton S virgins

The majority of the lines were obtained from Bloomington (http://flystocks.bio.indiana.edu/) or the Vienna Drosophila Resource Center (VDRC) stock center (http://stockcenter.vdrc.at) except where indicated otherwise.

### Behavioral Assays for *Drosophila melanogaster*

#### T-Maze Assay

The use of the T-maze assay is indicated in all figures with a fly head schematic with red-colored antennae to show that polyamine preference depends on olfactory sensory neurons on the antenna. 5–7 ds old flies raised at 25°C were used for all experiments, with the exception of experiments in which RNAi was used. In these experiments, experimental flies and genetic controls were raised at 30°C to enhance the effect of the RNAi. Flies were tested in groups of ~60 (30 females and 30 males or 60 females) in a T-maze and were allowed 1 min to respond to stimuli. Experimentation was carried out within climate-controlled boxes at 25°C and 60% rH in the dark. 50 μl of fresh odor solution at different concentrations diluted in distilled water applied on Whatman chromatography paper was provided in the odor tube, while 50 μl of distilled water (polyamine solvent) applied on Whatman chromatography paper was placed into the control tube. Unless otherwise indicated, 1 mM (~10 ppm according to measurements with a photo-ionization detector [PID]) of either putrescine or cadaverine were used. After experimentation, the number of flies in each tube was counted. An olfactory preference index (PI) was calculated by subtracting the number of flies on the test odor site from the number of flies on the control site and normalizing by the total number of flies. Statistical analysis was performed using two-way ANOVA and the Bonferroni multiple comparisons post-hoc test using Prism GraphPad 6.

#### Oviposition Assay

The oviposition assay is indicated in all figures by an illustration of the fly head with a red-labeled proboscis showing that oviposition preference depends on labellar taste neurons. In addition, oviposition assays are shown in simple schemes in the relevant figures. Here, the gray circle shows the oviposition plate filled with 1% agarose, and the colored squares indicate the addition of putrescine or cadaverine on one-half of the plate. Unless otherwise stated, 1 mM of polyamine was used in all assays. Mated female flies, reared on standard cornmeal medium at 25°C and 60% rH, were separated on ice from male flies at day 4 post-eclosion. Female flies were kept for 2 more days on fly food and used on day 6 for the oviposition assays. Flies raised at 25°C were used for all experiments, with the exception of experiments in which RNAi was used. In this experiments, experimental flies and genetic controls were raised at 30°C to enhance the effect of the RNAi. One percent low melting agarose was poured in a 60 x 15 mm petri-dish, and two halves were marked with a permanent marker on the bottom of the dish. Fifty μl of polyamine solution was applied on one side of the dish. In initial experiments, we also tested odor mixed into 1% low melting agarose compared to agarose only and obtained the same results as with applying the polyamine solution onto the hardened agarose. Sixty female flies were put in a gauzed top round cage, and the cage was closed with the test petri dish. Flies were kept for exactly 16 h in a light:dark cycle at controlled temperature and humidity conditions. An oviposition PI was calculated by subtracting the number of eggs on the test site from the number of eggs on the control site and normalized by the total number of eggs. Statistical analysis was performed using two-way ANOVA and the Bonferroni multiple comparisons post-hoc test using Prism GraphPad 6.

### Anatomy

Adult fly brains were dissected, fixed, and stained as described previously [60]. Briefly, brains were dissected in cold PBS, fixed with paraformaldehyde (2%, overnight at 4°C or for 2 h at RT), washed in PBS, 0.1% Triton X-100, 10% donkey serum and stained overnight at 4° C or for 2 h at RT with the primary and after washes in PBS, 0.1% Triton X-100 with the secondary antibody using the same conditions. For SPR staining, a procedure previously published was followed [15]. All microscopic observations were made at an Olympus FV-1000 confocal microscope or at a Leica MZ205 epifluorescence stereomicroscope. Images were processed using ImageJ and Photoshop. The following antibodies were used: chicken anti-GFP (molecular probes, 1:100), rabbit anti-Dsred (Clontech, Living colors DsRed polyclonal AB, 1:200), rat anti-N-cadherin (anti-N-cad DN-Ex #8, Developmental Studies Hybridoma Bank, 1:100), mouse anti-Dlarge (4F3-anti-discs large-c Developmental Studies Hybridoma Bank, 1:50), mouse anti-MIP (gift of C. Wegener, 1:50), rabbit anti-SPR ([15], gift of Y.-J. Kim, 1:10), rabbit anti-SPR (generated by H. Ammer, Ludwig Maximilians University Munich, Germany against the same peptide as used in [15]). Secondary antibodies used were: anti-chicken Alexa 488 (molecular probes, 1:250) and anti-rabbit Alexa 549 (molecular probes, 1:250), respectively.

MIP expression was analyzed using antibody staining with the aforementioned MIP antibody. All brains were processed at the same time using the same conditions. Images were taken at an Olympus FV-1000 confocal microscope at the exact same settings. Seven single confocal sections were selected over the entire volume of the antennal lobe without knowledge of the mating state. ROIs were drawn around the AL in each section, and image quantification was carried out blindly using FIJI ImageJ software. All MIP quantifications were normalized to the intensity of anti-Ncad staining of the same ROI of the same section. Statistical analysis (*t* test) and data illustration were carried out using Excel and GraphPad Prism software.

### In Vivo Calcium Imaging

For calcium imaging experiments, GCaMP6f (or for technical reasons GCaMP5 in experiments with SPR RNAi knockdown) were expressed under the control of IR41a-Gal4 or IR76b-Gal4. In vivo preparations of flies were prepared according to a method previously described [60]. In vivo preparations were imaged using a Leica DM6000FS fluorescent microscope equipped with a 40x water immersion objective and a Leica DFC360 FX fluorescent camera. All images were acquired with the Leica LAS AF E6000 image acquisition suit. Images were acquired for 20 s at a rate of 20 frames per second with 4 x 4 binning mode. During all measurements the exposure time was kept constant at 20 ms. For all experiments with odor stimulation, the stimulus was applied 5 s after the start of each measurement. A continuous and humidified airstream (2000 ml/min) was delivered to the fly throughout the experiment via an 8 mm diameter glass tube positioned 10 mm away from the preparation. A custom-made odor delivery system (Smartec, Martinsried), consisting of mass flow controllers (MFC) and solenoid valves, was used for delivering a continuous airstream and stimuli in all experiments. In all experiments, stimuli were delivered for 500 ms, and during stimulations the continuous flow was maintained at 2,000 ml/min. For putrescine stimulations, 1 ml of a precise concentration was filled in the odor delivery cup and the collected airspace odor was injected into the main airstream to give 0 ppm, 6 ppm, 8 ppm, and 10 ppm final concentrations for 500 ms without changing airstream strength. To measure the fluorescent intensity change, the region of interest was delineated by hand and the resulting time trace was used for further analysis. To calculate the normalized change in the relative fluorescence intensity, we used the following formula: ∆F/F = 100(Fn-F0)/F0, where Fn is the nth frame after stimulation and F0 is the averaged basal fluorescence of 15 frames before stimulation. The peak fluorescence intensity change is calculated as the mean of normalized trace over a 2 s time window during the stimulation period. The pseudo-colored images were generated in MATLAB using a custom written program. All analysis and statistical tests were done using Excel and GraphPad6 Prism software, respectively.

Imaging with taste stimuli was performed in a similar setup as described above, with some modifications. The flies expressing GCaMP-fluorescence under IR76b-Gal4 were prepared according to a method previously described [61]. The proboscis of the fly was pulled out by suction and fixed by gluing to prevent it from going back into the head capsule. For taste stimulation, taste stimuli were diluted in distilled water and delivered by a custom-built syringe delivery system to the proboscis. Distilled water (control), 1 mM, 10 mM, and 100 mM putrescine were applied, respectively. Application of the stimulus was monitored by a stereomicroscope. A drop of taste was delivered to touch the labellum. The stimulus was applied for 1 s after the start of each measurement. All analysis and statistical tests were done using Excel and GraphPad6 Prism software as described above.

### Quantitative PCR Analysis

Individual virgin female flies were mated with single males, observed, and separated after copulation. Two hundred of these mated females were kept for 4–6 h after mating following the same protocol as used for imaging. Antenna and brains of mated and virgin female flies of the same age were collected for RNA extraction. This procedure was repeated for three genetic replicates of 200 virgin and 200 mated flies. RNA was extracted using an RNA easy minikit (Qiagen) and used as a template for reverse transcription by superscript III reverse transcriptase (Invitrogen). Quantitative PCR was conducted using the following target gene primers: SPR (SPR-fwd: atgcacatcgtcagtagcct, SPR-rev: cagccgaccgaggaatatct) and MIP (MIP-fwd: ggacaatccgcacagcag, MIP-rev: ctgaacttgttccagccctg). H2A.Z, a histone variant (H2A.Z-fwd: tcgcatccatcgtcatctca, H2A.Z-rev: ctcggcggtcaggtattcc), was used as an internal control. All qPCR experiments were performed using the Applied Biosystems 7500 Fast Real-Time PCR system (Applied Biosystems). All amplifications were done using SYBR Green PCR Master Mix (Applied Biosystems). The thermal cycling conditions included an initial denaturation step at 95°C for 20 s, followed by 40 cycles at 95°C for 3 s and 60°C for 30 s. Melting curve analysis of every qPCR was conducted after each cycle. C_T_ (cycle threshold) values were used for analysis. The ∆∆C_T_ was calculated as previously described [62] by subtracting the control ∆C_T_ value of H2A.Z from the individual ∆C_T_ values of SPR and MIP for normalization (C_T_ mated–C_T_ virgin), respectively. The inverse logarithm was calculated to receive the expression fold change.

The numerical data used in all main and supplementary figures are included in S1 Data.

## Supporting Information

S1 Data The Excel spreadsheet contains, in separate sheets, the underlying numerical data and statistical analysis for the following figures with their relative panels: Fig 1, Fig 2, Fig 3, Fig 4, Fig 5, Fig 6, Fig 7, S1 Fig, S2 Fig, S3 Fig, S4 Fig, S5 Fig, S6 Fig and S7 Fig. (XLSX)

S1 Fig Polyamine behavior is modulated by mating state and SPR. (A) Graph shows number of eggs laid by Canton S mated and virgin females on agarose control (gray bars) or polyamine-rich substrates (putrescine: magenta, cadaverine: green) in 16 h oviposition assay. Number of eggs are averaged (*n* = 8± SEM, 60 ♀ flies/trial). (B) Average number of eggs laid by mated control and mated *Sex peptide receptor* mutant (*SPR*^*-/-*^) females on agarose control (gray bars) or polyamine-rich substrates (magenta/green) in the oviposition assay. Number of eggs are averaged (*n* = 8± SEM, 60 ♀ flies/trial). (C) Average number of eggs laid by controls and flies with knockdown of *SPR* in IR76b neurons (*IR76b-Gal4;UAS-SPRi*). Number of eggs are averaged (*n* = 8 ± SEM, 60 ♀ flies/trial). (D) Average number of eggs laid by controls and flies with knockdown of SPR in IR41a neurons (*IR41a-Gal4;UAS-SPRi*). Number of eggs are averaged (*n* = 8 ± SEM, 60 ♀ flies/trial). (E) Bar graph shows average number of eggs laid by controls and flies with re-expression of SPR in IR41a, IR76b, and GR66a neurons. Number of eggs are averaged (*n* = 8 ± SEM, 60 ♀ flies/trial).
(TIF)

S2 Fig IR76b is not expressed in ppk-positive neurons innervating the uterus. Expression analysis of IR76b compared to the ppk-Gal4 reporter in the female reproductive tract using *ppk-Gal4;UASmCD8GFP* (green in B and C), *IR76b-QF;QUASmdTomato-3xHa* (magenta in B and C), and *IR76b-Gal4;UASmCD8GFP* (green in D). Scale bars equal 200 μm. (A) Schematic drawing of the female reproductive tract showing the two ovaries, the snail-shaped seminal receptacle, the bilateral spermatheca and the uterus. (B–B′′) Epifluorescent images of reproductive organs. White arrow points to ppk-positive neurons innervating the uterus just underneath the seminal receptacle. Note that the uterus contains an egg in this case. Magenta staining has been overexposed and the color seen is primarily autofluorescence. (C–C′′) Magnified pictures of the boxed area in B of the same sample using confocal imaging. Tomato signal does not show positive cells but autofluorescence. (D) IR76b expression analysis with epifluorescence and confocal microscopy using the Gal4/UAS reporter system confirms the results obtained with the QF/QUAS system. The region of ppk-positive neurons beneath the seminal receptacle is devoid of GFP signal. The GFP signal does not show positive cells but autofluorescence.
(TIF)

S3 Fig The role of SP in the modulation of chemosensation. Bar graph shows average number of eggs laid by wild-type (wt) Canton S females mated to wild-type (wt) Canton S males and of wild-type (wt) Canton S females mated to Sex peptide mutant (*SP*^*0*^) males. Number of eggs is averaged (*n* = 8 ± SEM, 60 ♀ flies/trial).
(TIF)

S4 Fig MIP expression in the brain. (A) Representative pictures of virgin and mated female and mated male brains. MIP is expressed in neurons in the central brain as well as on axon tracts of peripheral neurons projecting into the brain (yellow arrowheads). Brains were stained with anti-MIP (yellow) and anti-NCad (blue). Images were taken at the confocal microscope.
(TIF)

S5 Fig MIPs are the putative central ligands for SPR. (A) Representative pictures of anti-MIP/anti-Dlarge antibody stained female brains showing knockdown of MIPs using two different RNAi lines and corresponding controls. The pan neuronal driver *elav-Gal4* removes MIP from all neurons. In brains from crosses with the specific driver *IR76b-Gal4* MIP staining is still present in most brain regions, but reduced in the antennal lobes (AL) and in the subesophageal zone (SEZ), where IR76b positive neurons project their axons. Upper panels show brain overview, while lower panels show substacks of the AL and SEZ, respectively. Images were taken at the confocal microscope. Scale bars equal 50 μm. (B) Average number of eggs laid by controls and flies with knockdown of three different MIPi transgenic lines in IR76b neurons (*IR76b-Gal4;UAS-MIPi*) on agarose control (gray bars) or polyamine-rich substrates (putrescine: magenta, cadaverine: green). Number of eggs are averaged (*n* = 8 ± SEM, 60 ♀ flies/trial).
(TIF)

S6 Fig Expression analysis of SPR using antibody staining. (A) Representative confocal images of brain and proboscis of *SPR*^*+/-*^ and *SPR*^*-/-*^ flies stained using two different SPR antibodies. No specific signal could be detected with either of the two antibodies. Arrows point to some staining in the SEZ and the labellum, which was also observed in the *SPR* mutants. Left panels show staining with a newly generated antibody against an SPR peptide (see Materials and Methods). Right panels show staining with a previously generated antibody [15]. Scale bars equal 50 μm. (B) Organization of the *SPR[Df(1)Exel6234] (SPR*^*-/-*^*)* deletion region on X chromosome. Df(1)Exel6234 covers the entire *SPR* gene and neighboring genomic regions. (C) Agarose gel electrophoresis of PCR product of a ~1.5 kb SPR gene fragment. *SPR[Df(1)Exel6234]* homozygous samples *(SPR*^*-/-*^*)* are negative for the *SPR* gene fragment amplification, showing that the *SPR* gene is deleted in those flies. By contrast, *SPR*^*+/-*^ and *SPR*^*+/+*^ flies show the expected band. (D) Agarose gel electrophoresis of PCR product of a ~500 bp region spanning the Df(1)Exel6234 deletion region. The band is visible in *SPR*^*-/-*^ homozygous and heterozygous samples, but not in *SPR*^*+/+*^ wildtype controls. (-)controls contain no genomic template DNA.
(TIF)

S7 Fig SPR and MIP expression modulates chemosensory behavior. (A) Confocal images showing MIP antibody staining (green) in seven representative single sections of the antennal lobe of a virgin and a mated female fly at 1–6 h post-mating. Sections were used for image quantification, and MIP staining intensity was normalized to staining intensity of anti-Ncad antibody staining (magenta) of the same section (see Materials and Methods). (B) Average number of eggs laid by control virgins and virgin females with overexpression of SPR or MIP under the control of the IR41a enhancer (*IR41a-Gal4*) or IR76b enhancer (*IR76b-Gal4*) on agarose control (gray bars) or polyamine-rich substrates (putrescine: magenta) in oviposition assays (*n* = 8). Number of eggs are averaged (*n* = 8 ± SEM, 60 ♀ flies/trial).
(TIF)

## Abbreviations

AL: antennal lobe
GPCR: G-protein coupled receptor
GRN: gustatory receptor neuron
IR: ionotropic receptor
MIP: myoinhibitory peptide
OSN: olfactory sensory neuron
PI: preference index
PID: photo-ionization detector
PLC: phospholipase C
PN: projection neuron
RNAi: RNA interference
ROI: region of interest
SEZ: subesophageal zone
sNPF: short neuropeptide F
sNPFR: short neuropeptide F receptor
SP: sex peptide
SPR: sex peptide receptor
VDRC: Vienna Drosophila Resource Center
