# Supplementary figures and images for: Neuropeptides Modulate Female Chemosensory Processing upon Mating in Drosophila
Source: PLoS Biol. 2016 May 4;14(5):e1002455. doi: 10.1371/journal.pbio.1002455 (PMC4856363; doi:10.1371/journal.pbio.1002455)

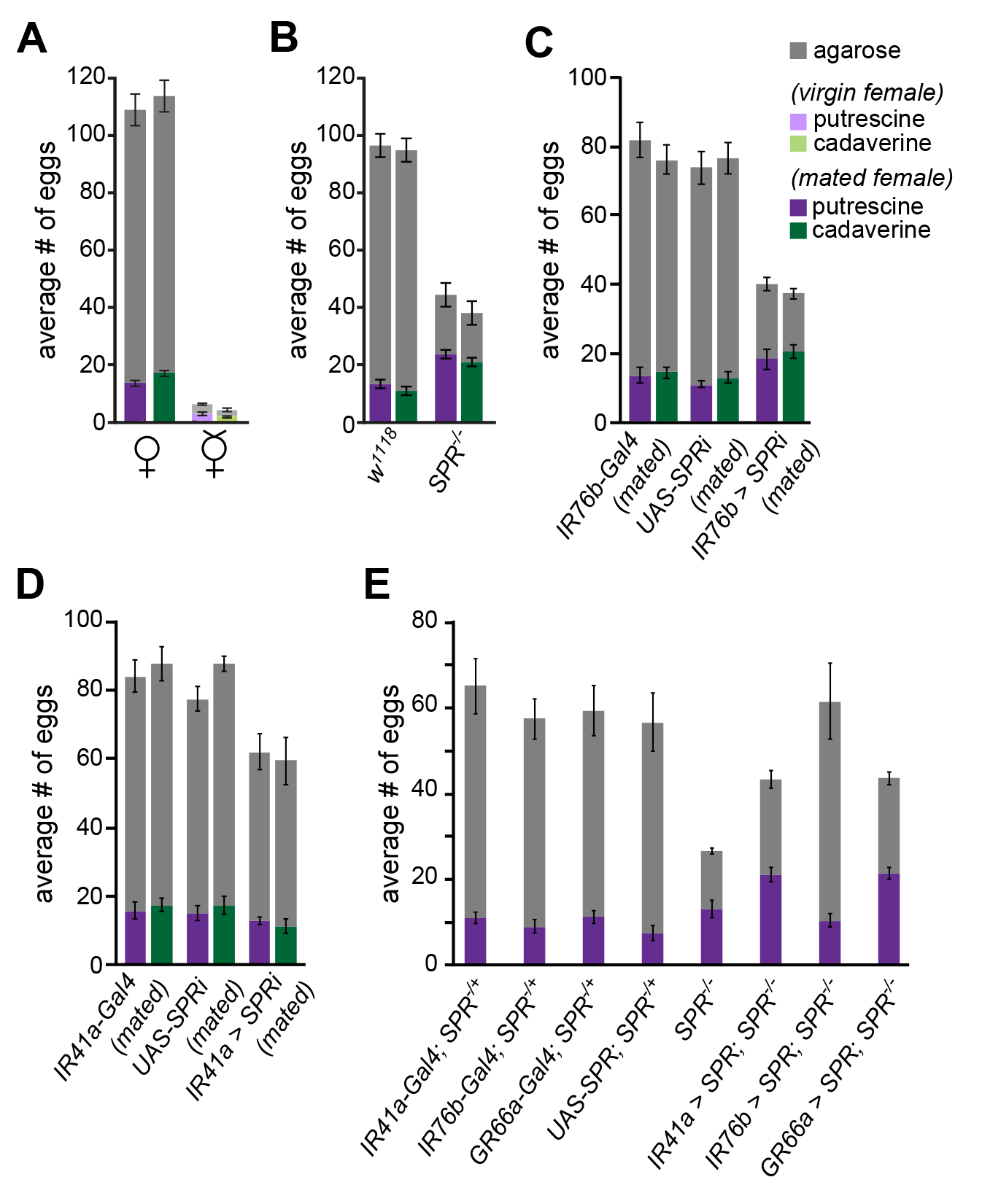

Supplement: S1 Fig — (A) Graph shows number of eggs laid by Canton S mated and virgin females on agarose control (gray bars) or polyamine-rich substrates (putrescine: magenta, cadaverine: green) in 16 h oviposition assay. Number of eggs are averaged (n = 8± SEM, 60 ♀ flies/trial). (B) Average number of eggs laid by mated control and mated Sex peptide receptor mutant (SPR-/-) females on agarose control (gray bars) or polyamine-rich substrates (magenta/green) in the oviposition assay. Number of eggs are averaged (n = 8± SEM, 60 ♀ flies/trial). (C) Average number of eggs laid by controls and flies with knockdown of SPR in IR76b neurons (IR76b-Gal4;UAS-SPRi). Number of eggs are averaged (n = 8 ± SEM, 60 ♀ flies/trial). (D) Average number of eggs laid by controls and flies with knockdown of SPR in IR41a neurons (IR41a-Gal4;UAS-SPRi). Number of eggs are averaged (n = 8 ± SEM, 60 ♀ flies/trial). (E) Bar graph shows average number of eggs laid by controls and flies with re-expression of SPR in IR41a, IR76b, and GR66a neurons. Number of eggs are averaged (n = 8 ± SEM, 60 ♀ flies/trial). (TIF) [file pbio.1002455.s002.tif]

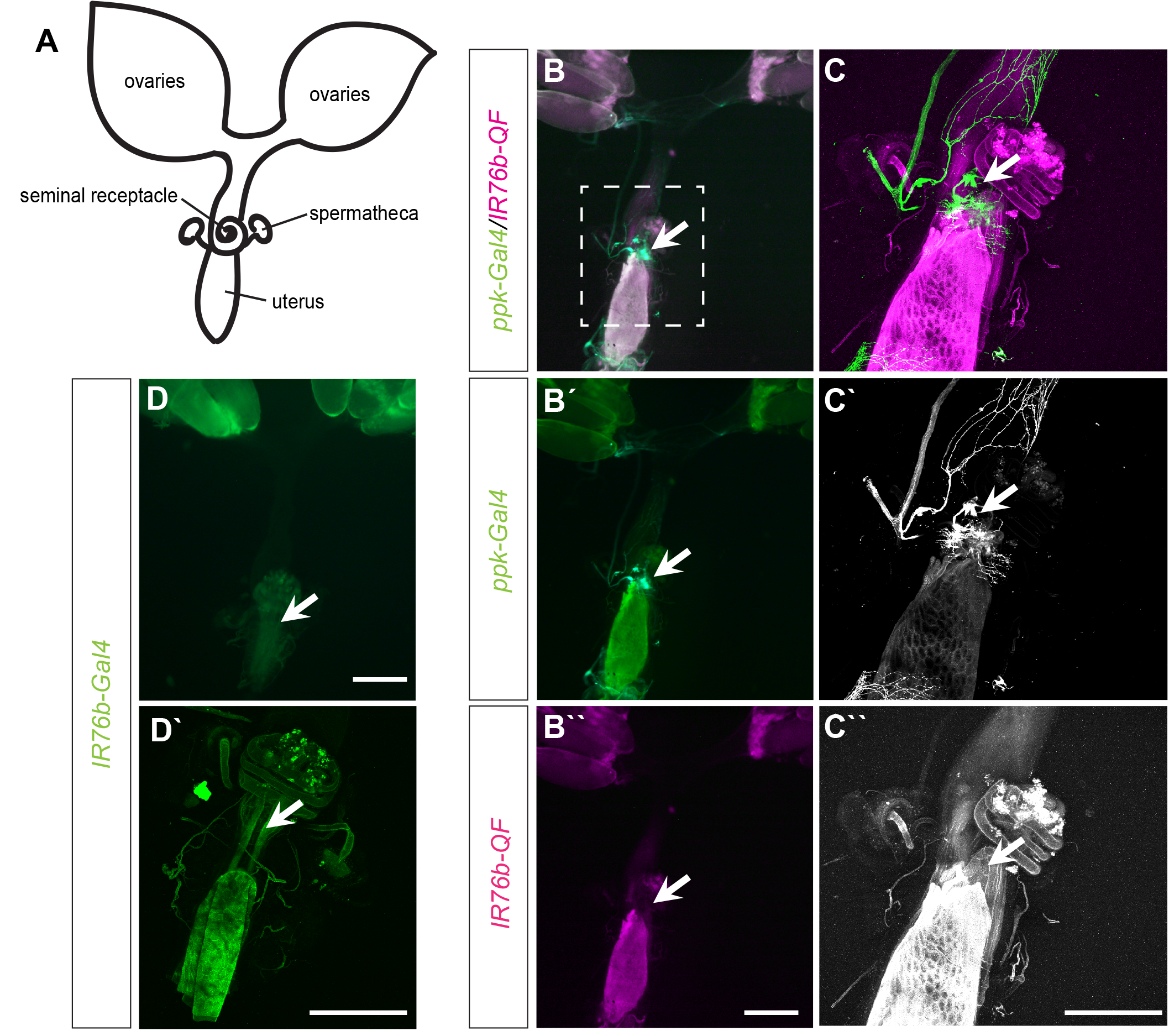

Supplement: S2 Fig — Expression analysis of IR76b compared to the ppk-Gal4 reporter in the female reproductive tract using ppk-Gal4;UASmCD8GFP (green in B and C), IR76b-QF;QUASmdTomato-3xHa (magenta in B and C), and IR76b-Gal4;UASmCD8GFP (green in D). Scale bars equal 200 μm. (A) Schematic drawing of the female reproductive tract showing the two ovaries, the snail-shaped seminal receptacle, the bilateral spermatheca and the uterus. (B–B′′) Epifluorescent images of reproductive organs. White arrow points to ppk-positive neurons innervating the uterus just underneath the seminal receptacle. Note that the uterus contains an egg in this case. Magenta staining has been overexposed and the color seen is primarily autofluorescence. (C–C′′) Magnified pictures of the boxed area in B of the same sample using confocal imaging. Tomato signal does not show positive cells but autofluorescence. (D) IR76b expression analysis with epifluorescence and confocal microscopy using the Gal4/UAS reporter system confirms the results obtained with the QF/QUAS system. The region of ppk-positive neurons beneath the seminal receptacle is devoid of GFP signal. The GFP signal does not show positive cells but autofluorescence. (TIF) [file pbio.1002455.s003.tif]

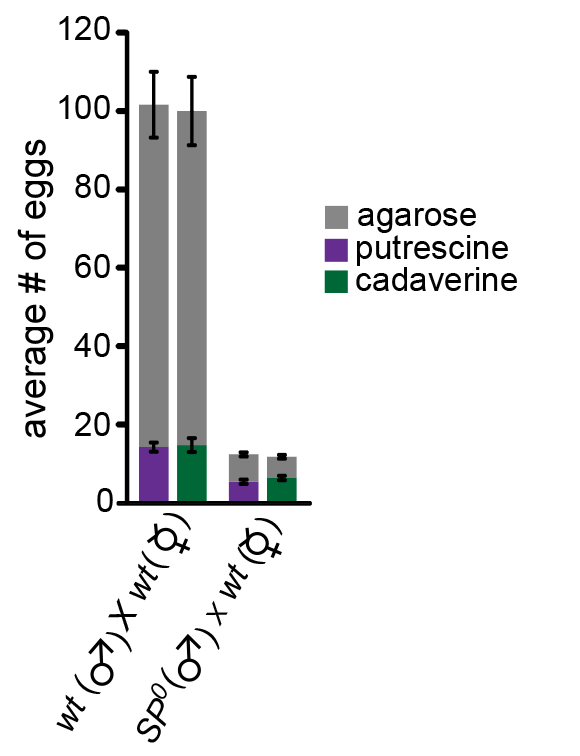

Supplement: S3 Fig — Bar graph shows average number of eggs laid by wild-type (wt) Canton S females mated to wild-type (wt) Canton S males and of wild-type (wt) Canton S females mated to Sex peptide mutant (SP0) males. Number of eggs is averaged (n = 8 ± SEM, 60 ♀ flies/trial). (TIF) [file pbio.1002455.s004.tif]

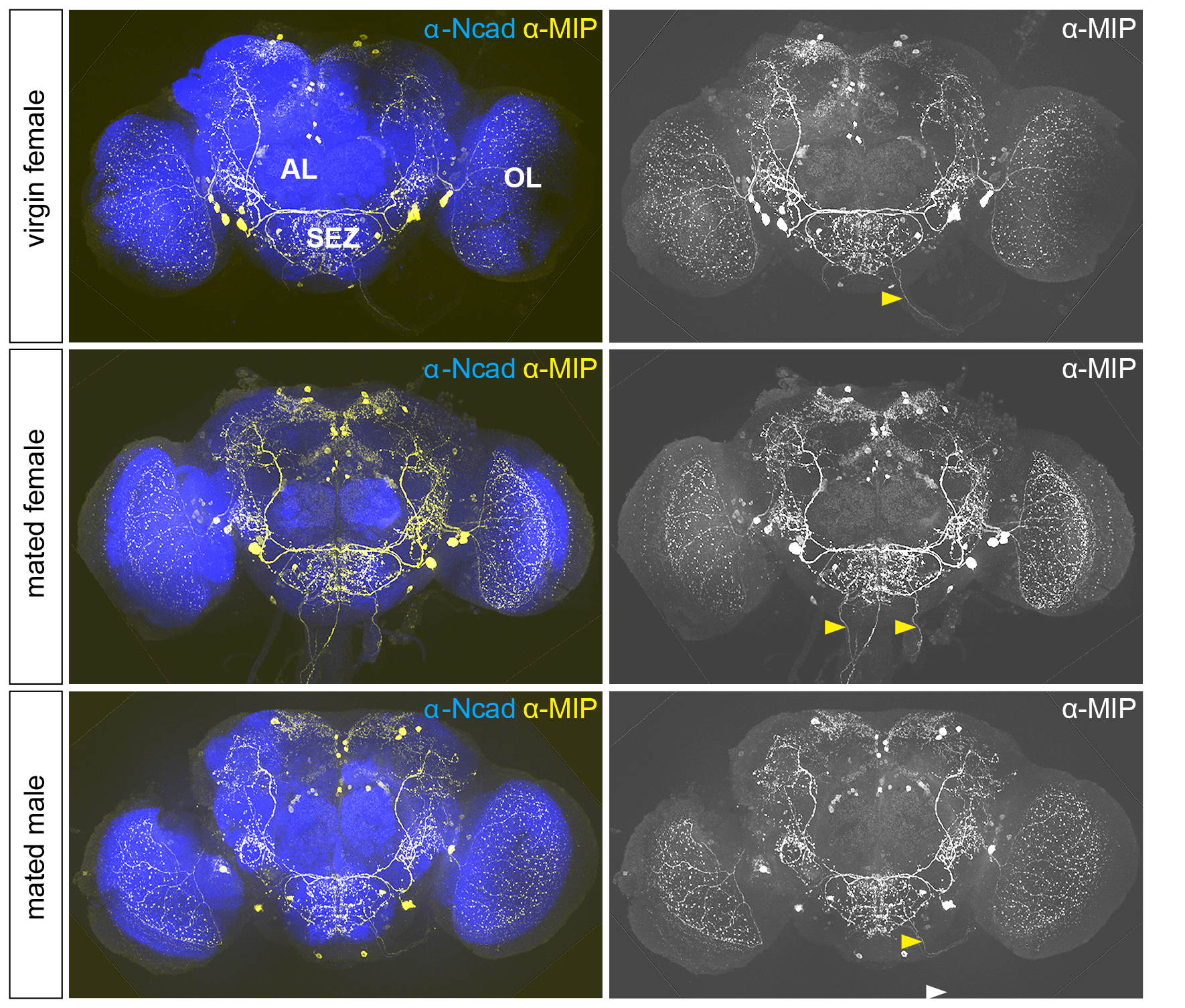

Supplement: S4 Fig — (A) Representative pictures of virgin and mated female and mated male brains. MIP is expressed in neurons in the central brain as well as on axon tracts of peripheral neurons projecting into the brain (yellow arrowheads). Brains were stained with anti-MIP (yellow) and anti-NCad (blue). Images were taken at the confocal microscope. (TIF) [file pbio.1002455.s005.tif]

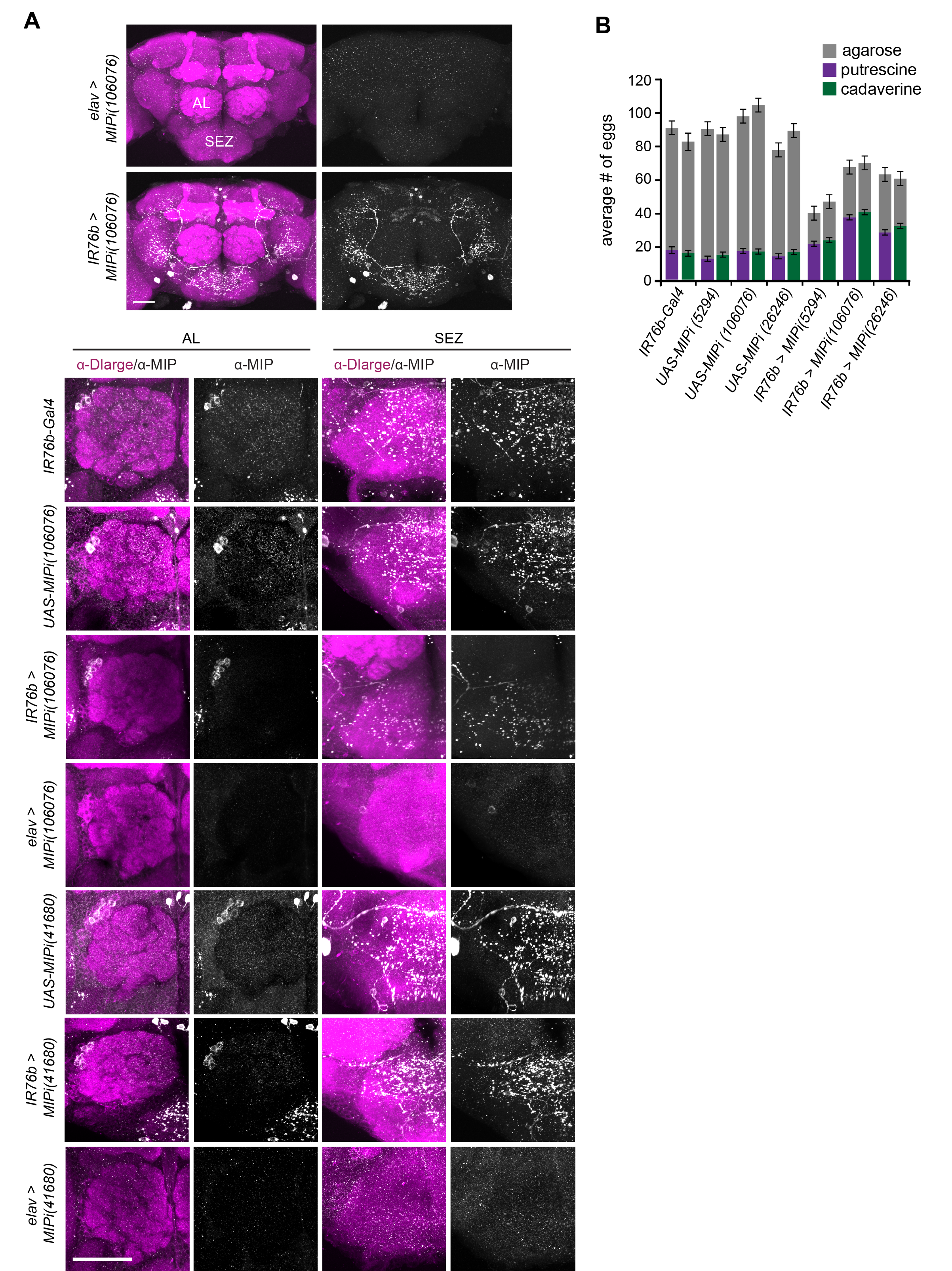

Supplement: S5 Fig — (A) Representative pictures of anti-MIP/anti-Dlarge antibody stained female brains showing knockdown of MIPs using two different RNAi lines and corresponding controls. The pan neuronal driver elav-Gal4 removes MIP from all neurons. In brains from crosses with the specific driver IR76b-Gal4 MIP staining is still present in most brain regions, but reduced in the antennal lobes (AL) and in the subesophageal zone (SEZ), where IR76b positive neurons project their axons. Upper panels show brain overview, while lower panels show substacks of the AL and SEZ, respectively. Images were taken at the confocal microscope. Scale bars equal 50 μm. (B) Average number of eggs laid by controls and flies with knockdown of three different MIPi transgenic lines in IR76b neurons (IR76b-Gal4;UAS-MIPi) on agarose control (gray bars) or polyamine-rich substrates (putrescine: magenta, cadaverine: green). Number of eggs are averaged (n = 8 ± SEM, 60 ♀ flies/trial). (TIF) [file pbio.1002455.s006.tif]

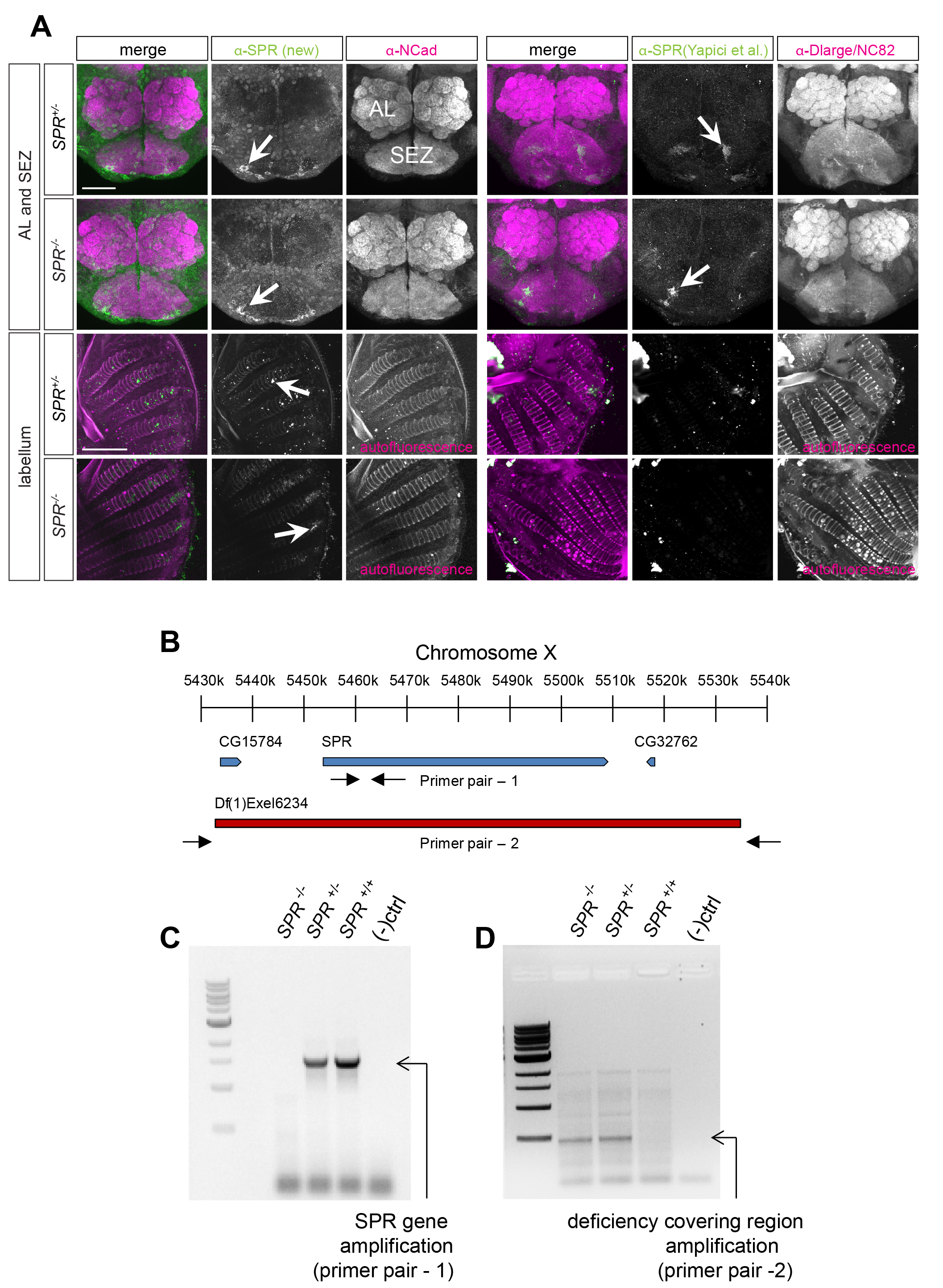

Supplement: S6 Fig — (A) Representative confocal images of brain and proboscis of SPR+/- and SPR-/- flies stained using two different SPR antibodies. No specific signal could be detected with either of the two antibodies. Arrows point to some staining in the SEZ and the labellum, which was also observed in the SPR mutants. Left panels show staining with a newly generated antibody against an SPR peptide (see Materials and Methods). Right panels show staining with a previously generated antibody [15]. Scale bars equal 50 μm. (B) Organization of the SPR[Df(1)Exel6234] (SPR-/-) deletion region on X chromosome. Df(1)Exel6234 covers the entire SPR gene and neighboring genomic regions. (C) Agarose gel electrophoresis of PCR product of a ~1.5 kb SPR gene fragment. SPR[Df(1)Exel6234] homozygous samples (SPR-/-) are negative for the SPR gene fragment amplification, showing that the SPR gene is deleted in those flies. By contrast, SPR+/- and SPR+/+ flies show the expected band. (D) Agarose gel electrophoresis of PCR product of a ~500 bp region spanning the Df(1)Exel6234 deletion region. The band is visible in SPR-/- homozygous and heterozygous samples, but not in SPR+/+ wildtype controls. (-)controls contain no genomic template DNA. (TIF) [file pbio.1002455.s007.tif]

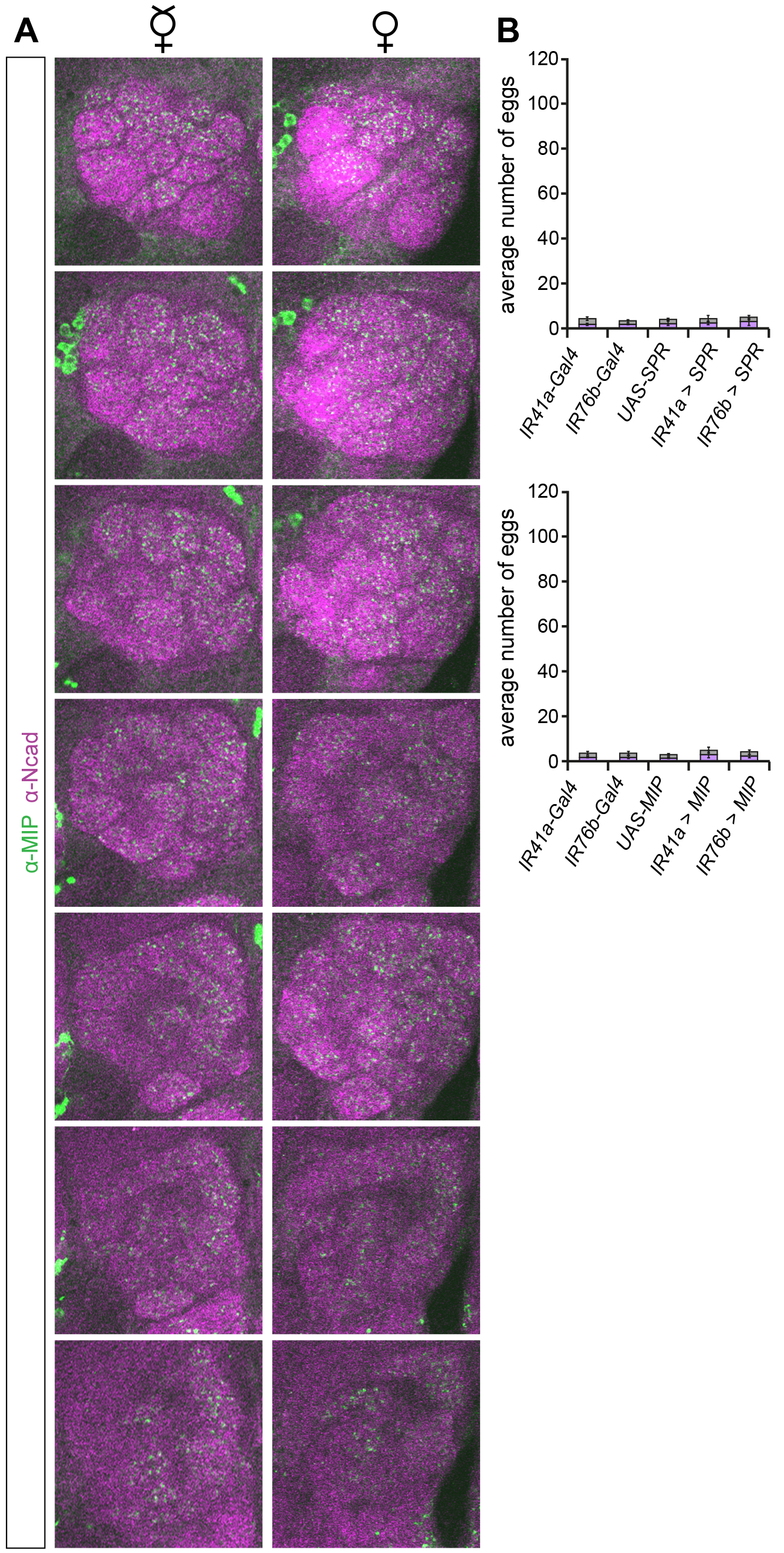

Supplement: S7 Fig — (A) Confocal images showing MIP antibody staining (green) in seven representative single sections of the antennal lobe of a virgin and a mated female fly at 1–6 h post-mating. Sections were used for image quantification, and MIP staining intensity was normalized to staining intensity of anti-Ncad antibody staining (magenta) of the same section (see Materials and Methods). (B) Average number of eggs laid by control virgins and virgin females with overexpression of SPR or MIP under the control of the IR41a enhancer (IR41a-Gal4) or IR76b enhancer (IR76b-Gal4) on agarose control (gray bars) or polyamine-rich substrates (putrescine: magenta) in oviposition assays (n = 8). Number of eggs are averaged (n = 8 ± SEM, 60 ♀ flies/trial). (TIF) [file pbio.1002455.s008.tif]
